# Interindividual Sleep Variability Across the Psychosis Spectrum: A Systematic Review and Meta-Analysis

**DOI:** 10.1001/jamanetworkopen.2026.24358

**Published:** 2026-07-22

**Authors:** Rosario Aronica, John Torous, Amedeo Minichino, Melinda Mills, Philip McGuire, Dominic Oliver

**Affiliations:** 1 Warneford Hospital, Oxford Health NHS Foundation Trust, Oxford, United Kingdom; 2 Department of Psychiatry, University of Oxford, Oxford, United Kingdom; 3 Department of Population Health, University of Oxford, Oxford, United Kingdom; 4 Beth Israel Deaconess Medical Center, Harvard Medical School, Boston, Massachusetts; 5 NIHR Oxford Biomedical Research Centre, Oxford, United Kingdom; 6 Leverhulme Centre for Demographic Science, University of Oxford, Oxford, United Kingdom; 7 Nuffield College, University of Oxford, Oxford, United Kingdom

## Abstract

**Importance:**

Sleep disturbances are common across the psychosis spectrum and may precede overt core clinical symptoms. Most studies focus on mean differences and overlook interindividual variability, which can be informative for stratified care.

**Objective:**

To quantify interindividual variability in actigraphy sleep measures across psychosis stages among individuals at clinical high risk for psychosis (CHR-P) and those with schizophrenia spectrum disorders (SSDs) compared with healthy control participants.

**Data Sources:**

The PubMed, Embase, MEDLINE, Cochrane Central Register of Controlled Trials, ClinicalTrials.gov, EU Clinical Trials Register, World Health Organization International Clinical Trials Registry Platform, and OpenGrey databases were searched for studies published from inception to April 29, 2024. An updated search was performed to identify studies published between April 30, 2024, and April 25, 2026.

**Study Selection:**

Case-control studies using wrist actigraphy in participants at CHR-P or with SSDs compared with healthy control participants were selected.

**Data Extraction and Synthesis:**

This systematic review and meta-analysis was preregistered with the Center for Open Science and followed the Preferred Reporting Items for Systematic Reviews and Meta-Analyses. Data were independently extracted under PRISMA standards. Two reviewers independently applied the inclusion criteria.

**Main Outcomes and Measures:**

The primary outcome was the natural logarithm of the variability ratio (lnVR) for total sleep time (TST). Secondary outcomes were the lnVR and the natural logarithm of the coefficient of variation ratio (lnCVR) for all actigraphy sleep parameters, including time in bed (TIB), sleep latency, wake after sleep onset (WASO), sleep efficiency, and number of awakenings. Variability metrics (lnVR and lnCVR) were pooled using random-effects models. Heterogeneity was assessed with *Q*, *I*^2^, and τ^2^ values and 95% CIs. Univariable mixed-effects meta-regression analysis tested antipsychotic use, with age and sex as moderators of the primary outcome. Each key result was tested against a panel of sensitivity analyses.

**Results:**

Of the 19 studies that met criteria, 18 entered the quantitative synthesis (1 study reported outcomes as medians and was excluded). Eighteen studies (n = 1358), including 202 individuals at CHR-P (mean [SD] age, 20.7 [2.8] years; 104 female [51.4%]), 574 individuals with SSDs (mean [SD] age, 37.7 [10.2] years; 363 female [63.2%]), and 582 healthy control participants (mean [SD] age, 31.5 [8.6] years; 311 male [53.4%]), were meta-analyzed. Participants at CHR-P had greater variability in TIB (lnVR, 0.29 [95% CI, 0.04-0.54] minutes; *I*^2^ = 0%; *P* = .02), WASO (0.45 [95% CI, 0.07-0.83] minutes; *I*^2^ = 83%; *P* = .02), and sleep efficiency (0.35% [95% CI, 0.06%-0.64%]; *I*^2^ = 72%; *P* = .02). Participants with SSDs had greater variability in TST (0.46 [95% CI, 0.28-0.64] minutes; *I*^2^ = 70%; *P* < .001), TIB (0.38 [95% CI, 0.19-0.56] minutes; *I*^2^ = 0%; *P* < .001), WASO (0.52 [95% CI, 0.15-0.89] minutes; *I*^2^ = 88%; *P* = .006), and sleep efficiency (0.27% [95% CI, 0.04%-0.50%]; *I*^2^ = 71%; *P* = .02). Moderator effects were nonsignificant.

**Conclusions and Relevance:**

In this systematic review and meta-analysis of 18 case-control studies, distinct digital phenotypes of sleep variability were identified among individuals across the psychosis spectrum compared with healthy control participants. These findings support further investigations of sleep as a candidate biomarker to inform stratified care in psychosis.

## Key Points

**Question:**

Is interindividual sleep variability greater in individuals across the psychosis spectrum than in healthy control participants?

**Findings:**

In this systematic review and meta-analysis of 1358 participants from 18 case-control studies, interindividual variability in time in bed, wake after sleep onset, and sleep efficiency was greater in those at clinical high risk for psychosis and those with schizophrenia spectrum disorders compared with healthy control participants. Total sleep time variability was significantly greater only in participants with schizophrenia spectrum disorders.

**Meaning:**

The distinct digital phenotypes of sleep variability across the psychosis spectrum described in this study support further investigations of sleep as a candidate biomarker for stratified care in psychosis.

## Teaser

This systematic review and meta-analysis uses actigraphy sleep measures to quantify interindividual sleep variability among individuals across the psychosis spectrum compared with healthy control participants.

## Introduction

Sleep disturbances are common across the psychosis spectrum, affecting up to 70% of individuals at clinical high risk for psychosis (CHR-P)^1,2^ and 80% of those with schizophrenia spectrum disorders (SSDs).^3^ In most of these studies, sleep was assessed with self-report questionnaires^4^ or polysomnography,^5^ which limits ecological validity. The advent of digital technologies, such as wrist actigraphy,^6^ enables continuous monitoring of sleep and offers a more scalable and powerful approach to characterize sleep disturbances in individuals with psychiatric conditions.^7^

**Figure 1.**
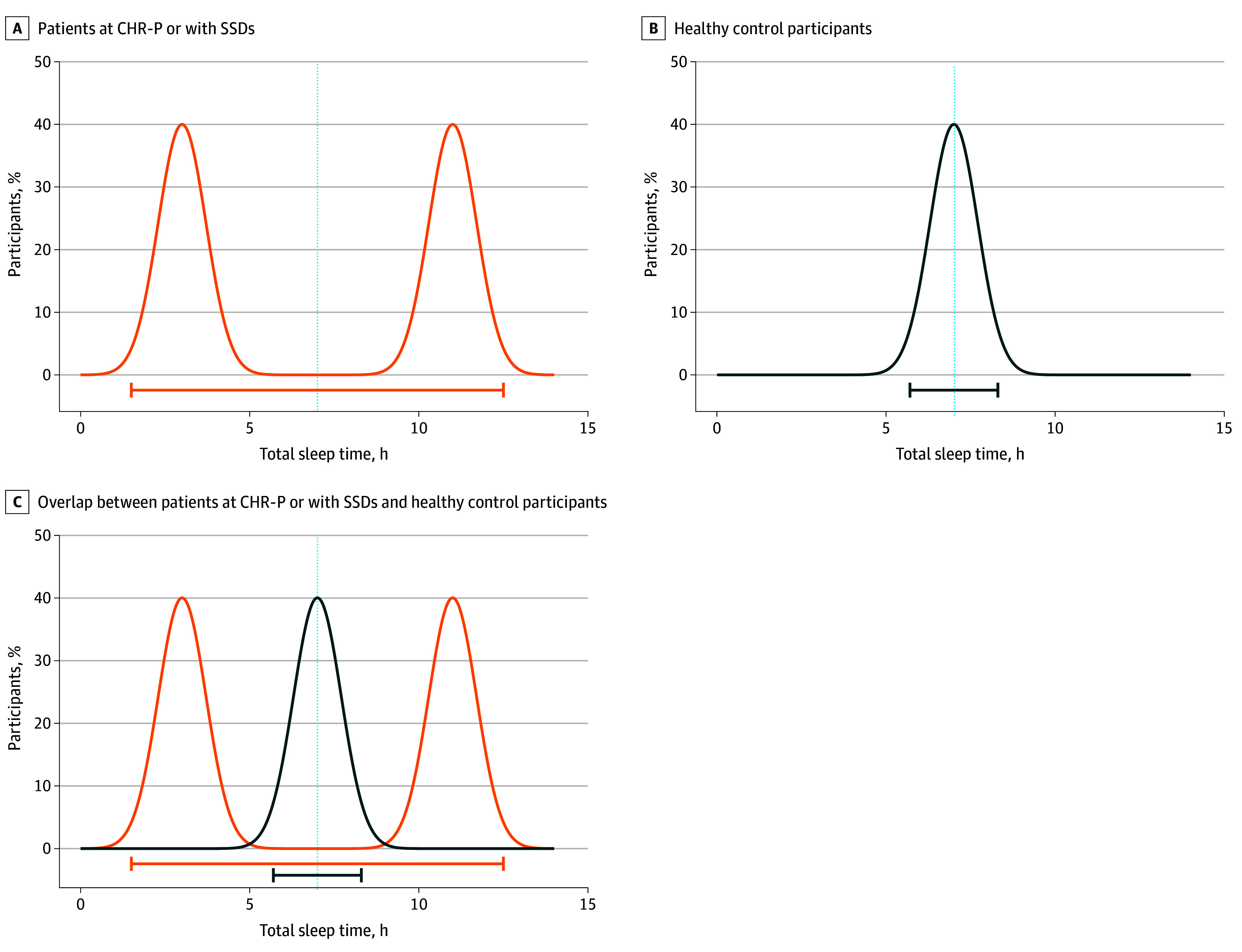
Line Graphs Showing Hypothetical Interindividual Variability in Total Sleep Time Among Individuals at Clinical High Risk for Psychosis (CHR-P) or With Schizophrenia Spectrum Disorders (SSDs) Compared With Healthy Control Participants Hypothetical distributions illustrate the study rationale. Participants at CHR-P or with SSDs are depicted with a conceptual bimodal distribution (A), reflecting short (approximately 3 hours) and long (approximately 11 hours) sleepers. In contrast, healthy control participants showed a unimodal distribution centered around approximately 7 hours of sleep (B), consistent with a normal pattern. Dashed vertical lines mark the mean, and horizontal lines with caps indicate the SD. While the mean values overlap (C), the bimodal distribution in the CHR-P or SSD group leads to higher SD.

To date, most wrist actigraphy studies in psychiatric populations have reported average alterations across patient samples.^8,9,10^ This approach assumes group-level homogeneity and may obscure clinically meaningful variability. While there are evident sleep disturbances in individuals at CHR-P and those with SSDs compared with control participants,^10^ these populations are highly heterogeneous in terms of clinical outcomes and underlying pathophysiology.^11^ Averaging across participants can therefore mask important interindividual differences within the same diagnostic group. For example, in a cohort comprising equal numbers of individuals with very short total sleep time (TST; approximately 3 hours) and very long TST (approximately 11 hours), the mean (approximately 7 hours) would resemble that of healthy control participants, concealing distributions with potentially distinct prognostic or therapeutic profiles (Figure 1). Failing to account for interindividual variability may therefore lead to null findings and overlook potential subpopulations with distinct sleep profiles.^12,13^

Two metrics from the meta-analysis of variation^14^ can quantify interindividual variability across studies: (1) the natural logarithm of the variability ratio (lnVR), which compares the SD of the clinical group with that of the control group, and (2) the natural logarithm of the coefficient of variation ratio (lnCVR), which additionally adjusts for between-group differences in the mean. Clinically, interindividual variability in sleep captures the extent to which individuals within the same diagnostic group differ from one another in their sleep, whether patients cluster tightly around a common sleep profile or instead span a wide range (eg, from severe insomnia to hypersomnia).

Despite these considerations, sleep variability remains underexplored in psychosis. Quantifying interindividual variability in sleep across the psychosis spectrum relative to healthy control participants could yield valuable insights into these conditions and provide a quantitative basis for future works on candidate subpopulations and stratified care.^14,15^

To address this gap, we conducted a systematic review and meta-analysis of case-control studies using wrist actigraphy to examine the interindividual variability in digital sleep phenotypes across psychosis stages (at CHR-P or with SSDs) compared with healthy control participants.

## Methods

This systematic review and meta-analysis aggregated deidentified data from previously published case-control studies. Ethical approval and informed consent were obtained by the original investigators of each included study. No additional institutional review board approval and no additional informed consent were required for this secondary meta-analysis. This study was preregistered with the Center for Open Science^16^ and followed the Preferred Reporting Items for Systematic Reviews and Meta-Analyses (PRISMA).

### Search Strategy and Study Selection

**Figure 2.**
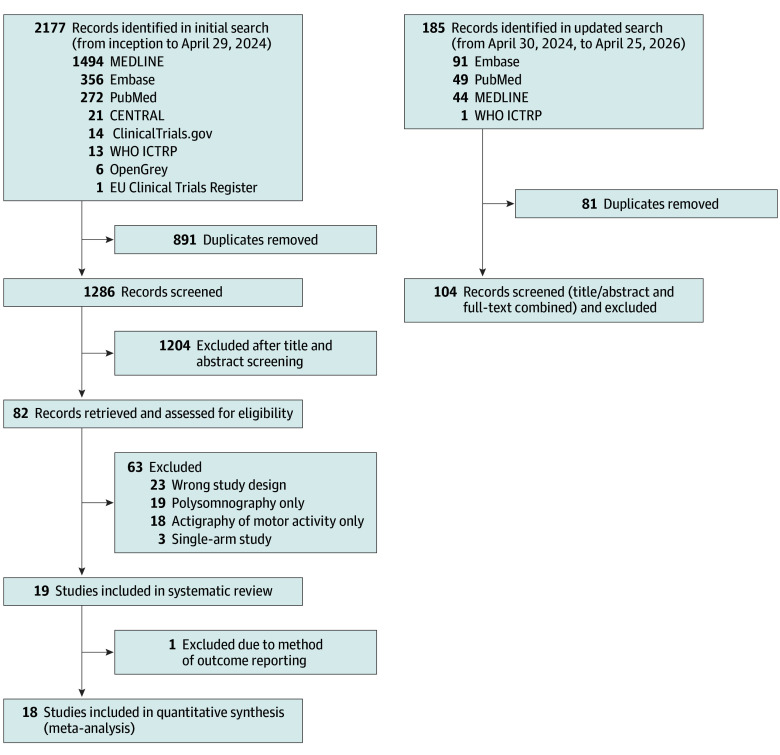
Study Flow Diagram CENTRAL indicates Cochrane Central Register of Controlled Trials; ICTRP, International Clinical Trials Registry Platform; WHO, World Health Organization.

This study extends prior work by our group on digital sleep phenotypes in CHR-P and SSDs, and it follows the same inclusion and exclusion criteria, data extraction protocols, and risk-of-bias assessments.^10^ The PubMed, Embase, MEDLINE, Cochrane Central Register of Controlled Trials, ClinicalTrials.gov, EU Clinical Trials Register, World Health Organization International Clinical Trials Registry Platform, and OpenGrey databases were searched from database inception to April 29, 2024, with no language restriction. The core search syntax combined *actigraphy* OR *actimetry* OR *wearable* OR *accelerometer* AND *psychosis* OR *schizophrenia* OR schizo* OR *clinical high risk* OR *prodromal* OR *ultra-high risk*; the full search syntax is reproduced in the eMethods in Supplement 1. The search strategy and study selection were last updated in April 2026 to identify records published from April 30, 2024, to April 25, 2026; 185 additional records were identified, none of which met the inclusion criteria (Figure 2).

Due to the high statistical heterogeneity encountered when pooling the CHR-P and SSD groups together, we decided to separate the analyses for each group. We included case-control studies that used wrist actigraphy to assess sleep in individuals at CHR-P or with SSDs and included a healthy control group. Each included study contributed effect sizes to one diagnostic comparison (CHR-P vs healthy control or SSDs vs healthy control); no study contributed to both comparisons. For studies with repeated assessments or multiple monitoring intervals, we selected the longest continuous actigraphy period to maximize the stability of habitual-sleep estimates, consistent with prior recommendations that recording windows of 7 days or longer improve test-retest reliability of actigraphy estimates.^17,18^

### Outcomes

Interindividual variability was primarily assessed with the log-transformed variability ratio (lnVR) of TST. Secondary outcomes included the log-transformed coefficient of variation ratio (lnCVR) for TST, as well as the lnVR and the lnCVR for the following additional actigraphy-derived sleep parameters: time in bed (TIB), sleep latency (SL), wake after sleep onset (WASO), sleep efficiency, and number of awakenings. lnVR was selected as the primary metric for its clinical interpretability and its ability to capture absolute variability differences linked to sleep instability; lnCVR provides complementary insight into relative variability.

### Risk of Bias

Risk of bias was assessed at the study level for the primary outcome using the ROBINS-E tool,^19^ by 2 independent reviewers (R.A., external reviewer), with disagreements resolved by team discussion. Per-study and per-outcome ratings are reported in eTable 1 in Supplement 1.

### Handling of Missing Data

Where SDs were not reported, they were calculated from the reported 95% CIs or SEs and the sample size, following the *Cochrane Handbook for Systematic Reviews of Interventions*.^20^ No study had imputed values for the variables of interest.

### Statistical Analysis

We fitted univariable random-effects meta-analyses with restricted maximum likelihood estimation of the between-study variance τ^2^ for each outcome and each diagnostic group (CHR-P vs healthy control and SSDs vs healthy control). Effect sizes (lnVR and lnCVR) were computed from the reported group means, SDs, and sample sizes; the inverse variance–weighted random-effects pool was the primary summary.^14^ Statistical heterogeneity was assessed with the *Q* statistic, *I*^2^, and τ^2^ values and CIs. Sensitivity subgroup analyses were also conducted by stratifying each primary or significant outcome by sampling epoch (≤30 seconds vs ≥60 seconds) and risk of bias, with the between-subgroup test computed as the omnibus moderator *Q* from a meta-regression on the stratifier.

Moderator analyses used univariable mixed-effects meta-regression to explore the influence of antipsychotics (percentage of clinical arm participants taking any antipsychotic), age, and sex on TST—the only 3 variables identified as significant moderators in our previous meta-analysis.^10^ Multivariable models were not fitted because the small number of studies per moderator (≤3-13) does not support stable multivariable estimation. All *P* values are 2-sided and statistical significance was set at .05. All analyses were conducted in R, version 4.5.1 (R Project for Statistical Computing), using the meta and metafor packages,^21^ and were last updated in April 2026.

## Results

**Figure 3.**
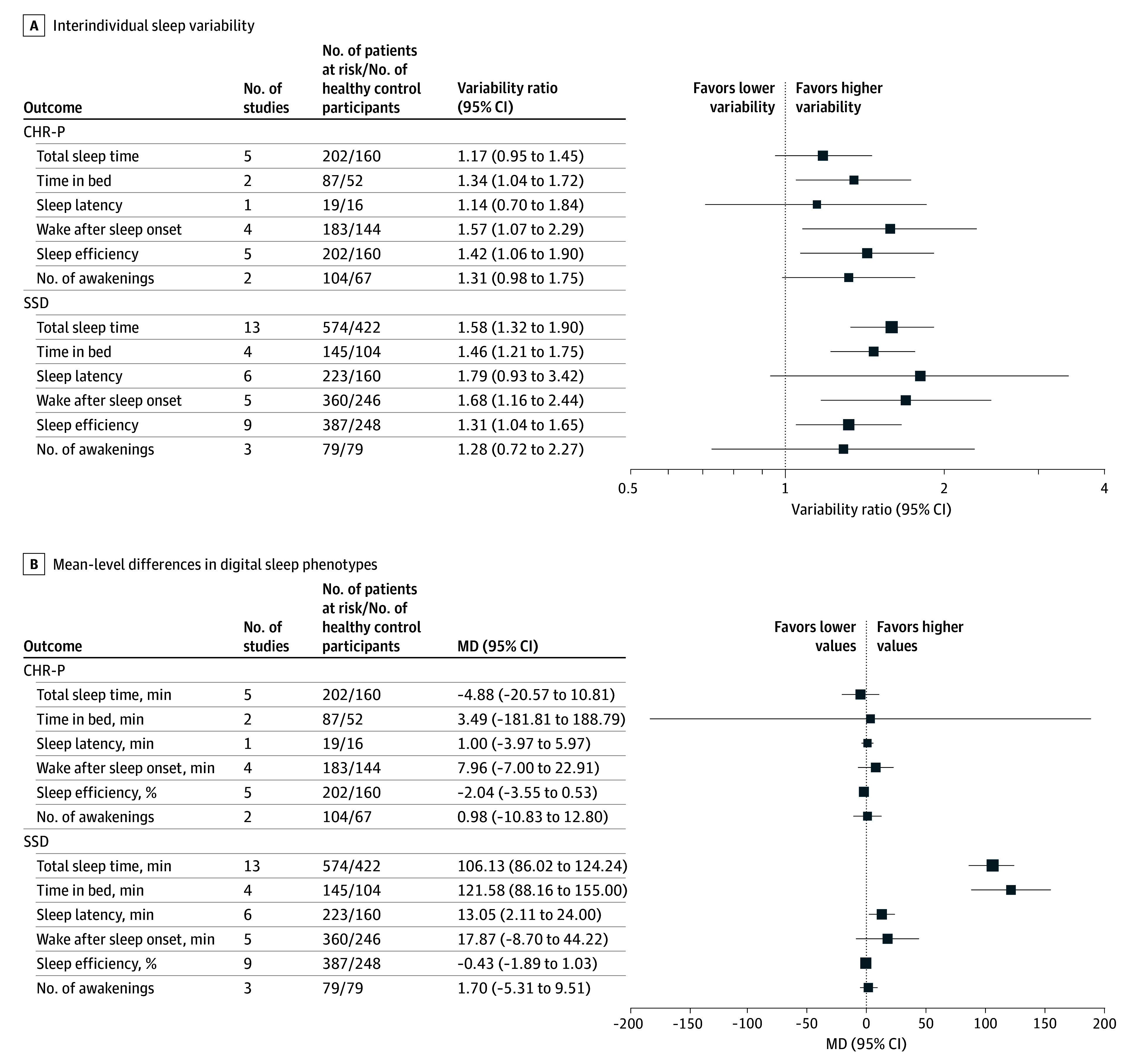
Forest Plots Showing Digital Sleep Phenotypes Across the Psychosis Spectrum A, Digital phenotypes of sleep variability by diagnostic subgroups of patients at clinical high risk for psychosis (CHR-P) or with schizophrenia spectrum disorders (SSDs), back-transformed from the natural log of variability ratio (lnVR). B, Digital sleep phenotypes for mean difference (MD) by diagnostic subgroup (from a previous meta-analysis by Aronica et al^10^). For each diagnostic subgroup, the squares represent the variability ratio or MD values for each sleep metric, with square size scaled to study weight; the error bars represent the 95% CIs.

Of the 19 studies meeting the inclusion criteria, 18 were included in the quantitative synthesis (1 study^22^ reported outcomes as medians and was excluded).^23,24,25,26,27,28,29,30,31,32,33,34,35,36,37,38,39,40^ The 18 studies contributed 1358 participants: 202 individuals at CHR-P (mean [SD] age, 20.7 [2.8] years; 104 female [51.4%] and 98 male [48.6%]), 574 with SSDs (mean [SD] age, 37.7 [10.2] years; 363 female [63.2%] and 211 male [36.8%]), and 582 healthy control participants (mean [SD] age, 31.5 [8.6] years; 217 female [46.6%] and 311 male [53.4%]). Of the 18 studies, 5 contributed a comparison of participants at CHR-P and healthy control participants^23,24,25,26,27^ and 13 contributed a comparison of participants with SSDs and healthy control participants^28,29,30,31,32,33,34,35,36,37,38,39,40^; no study contributed effect sizes to both diagnostic comparisons (each study appeared in only 1 of the 2 pairwise pools). Control participants were matched on age within each primary study (Table and eTable 2 in Supplement 1). Figure 3 illustrates digital sleep phenotypes by diagnostic subgroups of patients at CHR-P and with SSDs compared with healthy control participants.

**Table.** Sleep Variability and Mean-Level Effect Estimates in Participants at CHR-P and Those With SSDs vs Healthy Control Participants

Outcome	lnVR (95% CI)	lnCVR (95% CI)	MD (95% CI)^a^
Total sleep time, min			
CHR-P	0.16 (−0.05 to 0.37)	0.17 (−0.03 to 0.37)	−4.88 (−20.57 to 10.81)
SSD	0.46 (0.28-0.64)	0.22 (0.02-0.42)	106.13 (86.02-124.24)^b^
Time in bed, min			
CHR-P	0.29 (0.04-0.54)	0.27 (0.02-0.52)	3.49 (−181.81 to 188.79)
SSD	0.38 (0.19-0.56)	0.15 (−0.04 to 0.33)	121.58 (88.16-155.00)
Wake after sleep onset, min			
CHR-P	0.45 (0.07-0.83)	0.26 (−0.03 to 0.56)	7.96 (−7.00 to 22.91)
SSD	0.52 (0.15-0.89)	0.16 (0.03-0.29)	17.87 (−8.70 to 44.22)
Sleep latency, min			
CHR-P	0.13 (−0.36 to 0.61)	0.04 (−0.45 to 0.52)	1.00 (−3.97 to 5.97)
SSD	0.58 (−0.07 to 1.23)	0.05 (−0.28 to 0.38)	13.05 (2.11-24.00)
Sleep efficiency, %			
CHR-P	0.35 (0.06-0.64)	0.38 (0.08-0.67)	−2.04 (−3.55 to 0.53)
SSD	0.27 (0.04-0.50)	0.27 (0.03-0.51)	−0.43 (−1.89 to 1.03)
No. of awakenings			
CHR-P	0.27 (−0.02 to 0.56)	0.23 (−0.16 to 0.61)	0.98 (−10.83 to 12.80)
SSD	0.25 (−0.33 to 0.82)	−0.29 (−0.81 to 0.23)	1.70 (−5.31 to 9.51)

Abbreviations: CHR-P, clinical high risk for psychosis; lnCVR, natural logarithm of the coefficient of variation ratio; lnVR, natural logarithm of the variability ratio; MD, mean difference; SSD, schizophrenia spectrum disorder.

^a^ MD values are reproduced from our previous meta-analysis^10^ for comparison; lnVR and lnCVR values are new in the present analysis.

^b^ In participants with SSDs, the increase in mean total sleep time was associated with antipsychotic use (*R*^2^ = 88.14%), age (*R*^2^ = 38.89%), and sex (*R*^2^ = 26.29%) in our previous meta-analysis.

### Total Sleep Time

Individuals at CHR-P did not differ significantly from control participants in TST variability (lnVR, 0.16 [95% CI, –0.05 to 0.37] minutes; *I*^2^ = 47%; *P* = .14; and lnCVR, 0.17 [95% CI, –0.03 to 0.37] minutes; *I*^2^ =41%; *P* = .10) (eFigures 1 and 2 in Supplement 1). Moderator analyses of treatment with antipsychotic medications were associated with greater variability (β = 0.24 [95% CI, 0.06-0.41]; *R*^2^ = 100%; *P* = .008) (eFigure 3 in Supplement 1); given the small number of studies (*k* = 5), this finding should be interpreted as exploratory. Sex (β = 0.08 [95% CI, –0.15 to 0.30]; *R*^2^ = 0%; *P* = .50) (eFigure 4 in Supplement 1) and age (β = 0.11 [95% CI, –0.11 to 0.33]; *R*^2^ = 4.19%; *P* = .33) (eFigure 5 in Supplement 1) did not significantly moderate this effect.

In contrast, participants with SSDs showed greater TST variability than control participants (lnVR, 0.46 [95% CI, 0.28-0.64] minutes; *I*^2^ = 70%; *P* < .001) (eFigure 6 in Supplement 1). lnCVR values also suggested greater variability (0.22 [95% CI, 0.02-0.42] minutes; *I*^2^ = 76%; *P* = .03) (eFigure 7 in Supplement 1). No moderation effects were found for antipsychotic medications (β = –0.83 [95% CI, –2.13 to 0.48]; *R*^2^ = 0%; *P* = .21), sex (β = 1.20 [95% CI, –2.48 to 0.09]; *R*^2^ = 7.4%; *P* = .07), or age (β = 0.01 [95% CI, –0.01 to 0.03]; *R*^2^ = 0%; *P* = . 30) (eFigures 8, 9, and 10 in Supplement 1, respectively). In summary, interindividual TST variability was greater in participants with SSDs but not in those at CHR-P.

### Time in Bed

Participants at CHR-P showed increased TIB variability compared with healthy control participants (lnVR = 0.29 [95% CI, 0.04-0.54] minutes; *I*^2^ = 0%; *P* = .02; and lnCVR = 0.27 [95% CI, 0.02-0.52] minutes; *I*^2^ = 0%; *P* = .04) (eFigures 11 and 12 in Supplement 1). Participants with SSDs also had an increase in lnVR (0.38 [95% CI, 0.19-0.56] minutes; *I*^2^ = 0%; *P* < .001) (eFigure 13 in Supplement 1) but not in lnCVR (0.15 [95% CI, –0.04 to 0.33] minutes; *I*^2^ = 0%; *P* = .12) (eFigure 14 in Supplement 1). In summary, interindividual TIB variability was greater in both the CHR-P and SSD groups compared with control participants.

### SL and Number of Awakenings

No significant group differences were observed for SL. In participants at CHR-P, the lnVR was 0.13 minutes (95% CI, –0.36 to 0.61 minutes; *P* = .60) (eFigure 15 in Supplement 1) and the lnCVR was 0.04 minutes (95% CI, –0.45 to 0.52 minutes; *P* = .89) (eFigure 16 in Supplement 1). For participants with SSDs, the lnVR was 0.58 minutes (95% CI, –0.07 to 1.23 minutes; *I*^2^ = 94%; *P* = .08) (eFigure 17 in Supplement 1) and the lnCVR was 0.05 minutes (95% CI, –0.28 to 0.38 minutes; *I*^2^ = 78%; *P* = .77) (eFigure 18 in Supplement 1).

For number of awakenings, neither group showed differences compared with healthy control participants. In participants at CHR-P, the lnVR was 0.27 (95% CI, –0.02 to 0.56; *I*^2^ = 39%; *P* = .07) (eFigure 19 in Supplement 1) and the lnCVR was 0.23 (95% CI, –0.16 to 0.61; *I*^2^ = 66%; *P* = .25) (eFigure 20 in Supplement 1). In participants with SSDs, the lnVR was 0.25 (95% CI, –0.33 to 0.82]; *I*^2^ = 84%; *P* = .40) (eFigure 21 in Supplement 1) and the lnCVR was –0.29 (95% CI, –0.81 to 0.23; *I*^2^ =81%; *P* = .28) (eFigure 22 in Supplement 1). In summary, neither SL nor number of awakenings showed significant interindividual variability differences in the CHR-P group or SSD group.

### Wake After Sleep Onset

Both participants at CHR-P and those with SSDs had increased WASO variability. In those at CHR-P, the lnVR was 0.45 minutes (95% CI, 0.07-0.83 minutes; *I*^2^ = 83%; *P* = .02) (eFigure 23 in Supplement 1). In participants with SSDs, the lnVR was 0.52 minutes (95% CI, 0.15-0.89 minutes; *I*^2^ = 88%; *P* = .006) (eFigure 24 in Supplement 1), and the lnCVR was 0.16 minutes (95% CI, 0.03-0.29 minutes; *I*^2^ = 12%; *P* = .02) (eFigure 25 in Supplement 1).

### Sleep Efficiency

Individuals at CHR-P showed greater variability in sleep efficiency (lnVR, 0.35% [95% CI, 0.06%-0.64%]; *I*^2^ = 72%; *P* = .02; and lnCVR, 0.38% [95% CI, 0.08%-0.67%]; *I*^2^ = 73%; *P* = .01) (eFigures 26 and 27 in Supplement 1). Participants with SSDs also showed increased variability (lnVR, 0.27% [95% CI, 0.04%-0.50%]; *I*^2^ = 71%; *P* = .02; and lnCVR, 0.27% [95% CI, 0.03%-0.51%]; *I*^2^ = 73%; *P* = .03) (eFigures 28 and 29 in Supplement 1). In summary, both the CHR-P group and the SSD group showed greater interindividual variability in WASO and sleep efficiency than control participants.

### Sensitivity Analyses

Risk of bias was rated as high for 5 of 18 studies and as having some concerns for the remaining 13 (eTable 1 in Supplement 1); no study was rated low risk. Excluding the 5 studies at overall high risk of bias preserved the direction and statistical significance of all primary and significant outcomes (eTable 3 in Supplement 1). This suggests that residual bias from the 5 studies with high risk of bias is unlikely to explain the interindividual variability signal. Influence diagnostics (eFigures 30 and 31 in Supplement 1; summarized in eTable 4 in Supplement 1) showed that SSD findings (TST, TIB, WASO, and sleep efficiency) were robust to single-study omission, whereas CHR-P findings for WASO and sleep efficiency were sensitive to the omission of 2 studies—reflecting the small number of CHR-P analyses (*k* = 4-5). Stratification by sampling epoch (≤30 seconds vs ≥60 seconds) yielded estimates that were broadly comparable with the all-studies pools across the 4 primary or significant outcomes; full per-stratum estimates are reported in eTable 5 in Supplement 1. Small-study effects were assessed for the only outcome with at least 10 contributing studies (TST lnVR in participants with SSDs, *k* = 13), as recommended by Higgins et al.^20^ Results of the Egger regression test were not significant (intercept = 0.49 [95% CI, −0.06 to 1.05]; *P* = .89), suggesting no statistically detectable funnel plot asymmetry, although the test was moderately powered. The funnel plot and Egger scatter plot are provided in eFigure 32 in Supplement 1. The across-outcome summary is presented in eTable 6 in Supplement 1. Funnel plots for the 6 lower-*k* secondary outcomes are provided as visual inspection–only diagnostics in eFigure 33 in Supplement 1.

## Discussion

This is, to our knowledge, the first systematic review and meta-analysis evaluating sleep variability using wrist actigraphy studies in individuals at CHR-P and in those with SSDs. The findings suggest that both individuals at CHR-P and those with SSDs exhibit greater interindividual variability in TIB, WASO, and sleep efficiency compared with healthy control participants; only individuals with SSDs showed increased interindividual variability in TST compared with control participants. Whether the SSD-specific TST variability reflects progressive divergence of sleep duration with illness chronicity, or a fixed feature of established psychotic illness, requires longitudinal investigation.

Sleep disturbances are well characterized in individuals with psychotic disorders and may precede overt core clinical symptoms.^41^ In individuals at CHR-P, we identified increased interindividual variability in TIB, WASO, and sleep efficiency, which may reflect early disruptions in sleep regulation^42^ in at-risk individuals. Our previous meta-analysis showed no mean-level differences in TIB or WASO between those at CHR-P and healthy control participants, concealing this potentially informative signal. The increased variability in these measures may suggest distinct candidate at-risk subpopulations^43^ and support the view that disrupted sleep may not be merely secondary to psychotic symptoms but a candidate early biomarker of psychosis risk.^44^ Interestingly, the greater variability did not extend to TST,^45^ despite being increased in TIB. One interpretation is that individuals at CHR-P differ in the amount of time they spend in bed (some prolonging it, others shortening it) without corresponding changes in actual sleep duration. This dissociation between sleep opportunity and sleep obtained may represent an early feature of vulnerability,^46^ with increased variability in TST emerging only at later illness stages, as we observed in participants with SSDs.

In participants with SSDs, the variability profile resembled CHR-P but with the additional, statistically distinct increase in TST variability. In our previous mean-level meta-analysis,^10^ longer average TST in individuals with SSDs was associated with antipsychotic treatment. The present variance-level analysis shows that the increase in TST variability is not moderated by antipsychotic use; this is more consistent with candidate subpopulations who have SSDs with divergent baseline sleep disturbance (eg, some with hypersomnia, others with insomnia) on which antipsychotics exert a general tendency to lengthen TST.^47^ This pattern would also be consistent with a progressive disruption of sleep homeostatic mechanisms with illness progression.^13,48^

The use of wrist actigraphy in this meta-analysis^49^ underscores its translational potential for longitudinal research. Future studies should prioritize measures that were informative in this meta-analysis (WASO, TIB, sleep efficiency, and particularly TST) and collect continuous actigraphy data for at least 1 week to capture consistent sleep patterns.^17,18^ Combining actigraphy with ecological momentary assessments,^50^ clinical questionnaires,^51^ and passive smartphone data on sleep^52,53,54^ is essential to enrich the definition of sleep phenotypes and enhance early detection strategies.^55^ In this context, leveraging large, multisite datasets such as the AMP-SCZ (Accelerating Medicines Partnership Schizophrenia) program,^56^ will be crucial to validate these markers prospectively and study their potential prognostic and therapeutic relevance in clinical settings.

Randomized clinical trials and emulated target trials^57^ will also clarify whether interventions that stabilize sleep^58^ (eg, cognitive behavior therapy for insomnia,^59^ pharmacologic strategies,^60^ or chronotherapies^61,62^) reduce symptom severity, improve treatment response, and lower psychosis risk.^63,64^ For example, the SleepWell trial demonstrated that cognitive behavior therapy for sleep problems in individuals at CHR-P not only reduced insomnia severity but also lessened symptoms of depression, anxiety, and ideas of reference and persecution.^65^ Other interesting research avenues include novel mechanistic approaches to re-establish sleep homeostasis, such as mitochondria-targeted interventions.^66,67^

### Limitations

This study has some limitations. Because lnVR and lnCVR pool variances, they can inflate apparent interindividual variability if measurement noise differs systematically between groups. While residual measurement confounding cannot be excluded, each study used a single device and protocol for both clinical and control arms, so methodologic variance is shared between numerator and denominator within each study. A related consideration is that selection of the longest continuous actigraphy interval per study, while maximizing ecological validity, could introduce minor selection bias if recording duration differs systematically across clinical groups; reassuringly, stratification by sampling epoch (≤30 seconds vs ≥60 seconds) produced estimates broadly consistent with the all-studies pools across the 4 primary or significant outcomes, suggesting these methodologic factors did not materially confound the findings.

The case-control design of the included studies precludes causal inference and prognostic claims; therefore, we cannot determine whether elevated interindividual sleep variability precedes clinical change, and prospective studies—in CHR-P samples followed to first-episode psychosis, and in SSD samples followed to relapse—will be needed to address this. Between-study heterogeneity and sample composition imposes further constraints on generalizability: race and ethnicity were not consistently reported in the primary studies, and the CHR-P literature draws on heterogeneous instruments (Comprehensive Assessment of At-Risk Mental States [CAARMS], Structured Interview for Psychosis-Risk Syndromes [SIPS], and Community Assessment of Psychic Experiences [CAPE]) and inclusion criteria (genetic risk and psychotic-like experiences), limiting harmonization.

Statistical power was a further constraint, particularly for moderator analyses in SSDs: at most, 13 SSD studies contributed to the most-populated moderator, with fewer providing usable covariate data on antipsychotic use (with dose unavailable), age, or sex, so null moderator effects in this group should be interpreted cautiously. Finally, because our analyses operate on aggregated study-level statistics, the findings establish increased interindividual variability rather than the existence of discrete clinical subgroups. Individual-participant data are required to determine whether the observed dispersion reflects identifiable subpopulations or a single broader distribution.

## Conclusions

In this systematic review and meta-analysis of 18 case-control studies, we identified distinct digital phenotypes of sleep variability among individuals across the psychosis spectrum compared with healthy control participants. Both individuals at CHR-P and those with SSDs showed greater variability in TIB, WASO, and sleep efficiency, while only those with SSDs showed greater TST variability. These findings support further investigations of sleep as a candidate biomarker to inform stratified care in psychosis^68^ using individual-participant data and prospective studies.

## Notes

Supplement 1. **eMethods.** Full Search Strategy and Database Syntax **eTable 1.** ROBINS-E Ratings Per Study and Per Domain (Primary Outcome) **eTable 2.** Characteristics of Included Studies **eTable 3.** Sensitivity Meta-Analysis Excluding Studies at Overall High Risk of Bias **eTable 4.** Leave-One-Out Sensitivity Summary for the Primary or Significant Outcomes (lnVR Only) **eTable 5.** Subgroup Analyses Stratified by Sampling Epoch **eTable 6.** Small-Study Effects/Publication-Bias Assessment Across the Primary and Significant Outcomes **eFigure 1.** Total Sleep Time (TST)—Patients With Clinical High Risk for Psychosis (CHR-P): Natural Logarithm of the Variability Ratio (lnVR) (*k* = 5; Restricted Maximum Likelihood [REML]) **eFigure 2.** Total Sleep Time—CHR-P: lnCVR (*k* = 5; REML) **eFigure 3.** Total Sleep Time—CHR-P: Meta-Regression by Antipsychotic Use **eFigure 4.** Total Sleep Time—CHR-P: Meta-Regression by Sex **eFigure 5.** Total Sleep Time—CHR-P: Meta-Regression by Age **eFigure 6.** Total Sleep Time—Patients With Schizophrenia Spectrum Disorders (SSD): lnVR (*k* = 13; REML) **eFigure 7.** Total Sleep Time—SSD: lnCVR (*k* = 13; REML) **eFigure 8.** Total Sleep Time—SSD: Meta-Regression by Antipsychotic Use **eFigure 9.** Total Sleep Time—SSD: Meta-Regression by Sex **eFigure 10.** Total Sleep Time—SSD: Meta-Regression by Age **eFigure 11.** Time in Bed (TIB)—CHR-P: lnVR (*k* = 2; REML) **eFigure 12.** Time in Bed—CHR-P: lnCVR (*k* = 2; REML) **eFigure 13.** Time in Bed—SSD: lnVR (*k* = 4; REML) **eFigure 14.** Time in Bed—SSD: lnCVR (*k* = 4; REML) **eFigure 15.** Sleep Latency (SL)—CHR-P: lnVR (*k* = 1) **eFigure 16.** Sleep Latency—CHR-P: lnCVR (*k* = 1) **eFigure 17.** Sleep Latency—SSD: lnVR (*k* = 6; REML) **eFigure 18.** Sleep Latency—SSD: lnCVR (*k* = 6; REML) **eFigure 19.** Number of Awakenings (AWK)—CHR-P: lnVR (*k* = 2; REML) **eFigure 20.** Number of Awakenings—CHR-P: lnCVR (*k* = 2; REML) **eFigure 21.** Number of Awakenings—SSD: lnVR (*k* = 3; REML) **eFigure 22.** Number of Awakenings—SSD: lnCVR (*k* = 3; REML) **eFigure 23.** Wake After Sleep Onset (WASO)—CHR-P: lnVR (*k* = 4; REML) **eFigure 24.** Wake After Sleep Onset—SSD: lnVR (*k* = 5; REML) **eFigure 25.** Wake After Sleep Onset—SSD: lnCVR (*k* = 5; REML) **eFigure 26.** Sleep Efficiency (SE)—CHR-P: lnVR (*k* = 5; REML) **eFigure 27.** Sleep Efficiency—CHR-P: lnCVR (*k* = 5; REML) **eFigure 28.** Sleep Efficiency—SSD: lnVR (*k* = 9; REML) **eFigure 29.** Sleep Efficiency—SSD: lnCVR (*k* = 9; REML) **eFigure 30.** Leave-One-Out Sensitivity Analyses for Total Sleep Time (TST), Time in Bed (TIB), Wake After Sleep Onset (WASO), and Sleep Efficiency (SE) in Patients With Clinical High Risk for Psychosis (CHR-P) and Schizophrenia Spectrum Disorders (SSD) **eFigure 31.** Baujat Plots for Total Sleep Time (TST), Time in Bed (TIB), Wake After Sleep Onset (WASO), and Sleep Efficiency (SE) in Patients With Clinical High Risk for Psychosis (CHR-P) and Schizophrenia Spectrum Disorders (SSD) **eFigure 32.** Funnel Plot and Egger Regression for the Primary Outcome (Total Sleep Time [TST] lnVR) in Patients With Schizophrenia Spectrum Disorders (SSD) **eFigure 33.** Funnel Plots for Key Secondary Outcomes (Time in Bed [TIB], Wake After Sleep Onset [WASO], and Sleep Efficiency [SE]) in Patients With Clinical High Risk for Psychosis (CHR-P) and Schizophrenia Spectrum Disorders (SSD)
eReferences

Supplement 2.
Data Sharing Statement
