# Supplementary material for: Interindividual Sleep Variability Across the Psychosis Spectrum: A Systematic Review and Meta-Analysis
Source: JAMA Netw Open. 2026 Jul 22;9(7):e2624358. doi: 10.1001/jamanetworkopen.2026.24358 (PMC13392811; doi:10.1001/jamanetworkopen.2026.24358)
Supplement: Supplement 1. — eMethods. Full Search Strategy and Database Syntax eTable 1. ROBINS-E Ratings Per Study and Per Domain (Primary Outcome) eTable 2. Characteristics of Included Studies eTable 3. Sensitivity Meta-Analysis Excluding Studies at Overall High Risk of Bias eTable 4. Leave-One-Out Sensitivity Summary for the Primary or Significant Outcomes (lnVR Only) eTable 5. Subgroup Analyses Stratified by Sampling Epoch eTable 6. Small-Study Effects/Publication-Bias Assessment Across the Primary and Significant Outcomes eFigure 1. Total Sleep Time (TST)—Patients With Clinical High Risk for Psychosis (CHR-P): Natural Logarithm of the Variability Ratio (lnVR) (k = 5; Restricted Maximum Likelihood [REML]) eFigure 2. Total Sleep Time—CHR-P: lnCVR (k = 5; REML) eFigure 3. Total Sleep Time—CHR-P: Meta-Regression by Antipsychotic Use eFigure 4. Total Sleep Time—CHR-P: Meta-Regression by Sex eFigure 5. Total Sleep Time—CHR-P: Meta-Regression by Age eFigure 6. Total Sleep Time—Patients With Schizophrenia Spectrum Disorders (SSD): lnVR (k = 13; REML) eFigure 7. Total Sleep Time—SSD: lnCVR (k = 13; REML) eFigure 8. Total Sleep Time—SSD: Meta-Regression by Antipsychotic Use eFigure 9. Total Sleep Time—SSD: Meta-Regression by Sex eFigure 10. Total Sleep Time—SSD: Meta-Regression by Age eFigure 11. Time in Bed (TIB)—CHR-P: lnVR (k = 2; REML) eFigure 12. Time in Bed—CHR-P: lnCVR (k = 2; REML) eFigure 13. Time in Bed—SSD: lnVR (k = 4; REML) eFigure 14. Time in Bed—SSD: lnCVR (k = 4; REML) eFigure 15. Sleep Latency (SL)—CHR-P: lnVR (k = 1) eFigure 16. Sleep Latency—CHR-P: lnCVR (k = 1) eFigure 17. Sleep Latency—SSD: lnVR (k = 6; REML) eFigure 18. Sleep Latency—SSD: lnCVR (k = 6; REML) eFigure 19. Number of Awakenings (AWK)—CHR-P: lnVR (k = 2; REML) eFigure 20. Number of Awakenings—CHR-P: lnCVR (k = 2; REML) eFigure 21. Number of Awakenings—SSD: lnVR (k = 3; REML) eFigure 22. Number of Awakenings—SSD: lnCVR (k = 3; REML) eFigure 23. Wake After Sleep Onset (WASO)—CHR-P: lnVR (k = 4; REML) eFigure 24. Wake [file jamanetwopen-e2624358-s001.pdf]

Supplementary Online Content

Aronica R, Torous J, Minichino A, Mills M, McGuire P, Oliver D. Interindividual sleep variability across the psychosis spectrum: a systematic review and meta-analysis. *JAMA Netw Open*. 2026;9(7):e2624358. doi:10.1001/jamanetworkopen.2026.24358

eMethods. Full Search Strategy and Database Syntax

eTable 1. ROBINS-E Ratings Per Study and Per Domain (Primary Outcome)

eTable 2. Characteristics of Included Studies

eTable 3. Sensitivity Meta-Analysis Excluding Studies at Overall High Risk of Bias

eTable 4. Leave-One-Out Sensitivity Summary for the Primary or Significant Outcomes (lnVR Only)

eTable 5. Subgroup Analyses Stratified by Sampling Epoch

eTable 6. Small-Study Effects/Publication-Bias Assessment Across the Primary and Significant Outcomes

eFigure 1. Total Sleep Time (TST)—Patients With Clinical High Risk for Psychosis (CHR-P): Natural Logarithm of the Variability Ratio (lnVR) (*k* = 5; Restricted Maximum Likelihood [REML])

eFigure 2. Total Sleep Time—CHR-P: lnCVR (*k* = 5; REML)

eFigure 3. Total Sleep Time—CHR-P: Meta-Regression by Antipsychotic Use

eFigure 4. Total Sleep Time—CHR-P: Meta-Regression by Sex

eFigure 5. Total Sleep Time—CHR-P: Meta-Regression by Age

eFigure 6. Total Sleep Time—Patients With Schizophrenia Spectrum Disorders (SSD): lnVR (*k* = 13; REML)

eFigure 7. Total Sleep Time—SSD: lnCVR (*k* = 13; REML)

eFigure 8. Total Sleep Time—SSD: Meta-Regression by Antipsychotic Use

eFigure 9. Total Sleep Time—SSD: Meta-Regression by Sex

eFigure 10. Total Sleep Time—SSD: Meta-Regression by Age

eFigure 11. Time in Bed (TIB)—CHR-P: lnVR (*k* = 2; REML)

eFigure 12. Time in Bed—CHR-P: lnCVR (*k* = 2; REML)

eFigure 13. Time in Bed—SSD: lnVR (*k* = 4; REML)

eFigure 14. Time in Bed—SSD: lnCVR (*k* = 4; REML)

eFigure 15. Sleep Latency (SL)—CHR-P: lnVR (*k* = 1)

eFigure 16. Sleep Latency—CHR-P: lnCVR (*k* = 1)

eFigure 17. Sleep Latency—SSD: lnVR (*k* = 6; REML)

eFigure 18. Sleep Latency—SSD: lnCVR (*k* = 6; REML)

© 2026 Aronica R et al. *JAMA Network Open*.

eFigure 19. Number of Awakenings (AWK)—CHR-P: lnVR (*k* = 2; REML)

eFigure 20. Number of Awakenings—CHR-P: lnCVR (*k* = 2; REML)

eFigure 21. Number of Awakenings—SSD: lnVR (*k* = 3; REML)

eFigure 22. Number of Awakenings—SSD: lnCVR (*k* = 3; REML)

eFigure 23. Wake After Sleep Onset (WASO)—CHR-P: lnVR (*k* = 4; REML)

eFigure 24. Wake After Sleep Onset—SSD: lnVR (*k* = 5; REML)

eFigure 25. Wake After Sleep Onset—SSD: lnCVR (*k* = 5; REML)

eFigure 26. Sleep Efficiency (SE)—CHR-P: lnVR (*k* = 5; REML)

eFigure 27. Sleep Efficiency—CHR-P: lnCVR (*k* = 5; REML)

eFigure 28. Sleep Efficiency—SSD: lnVR (*k* = 9; REML)

eFigure 29. Sleep Efficiency—SSD: lnCVR (*k* = 9; REML)

eFigure 30. Leave-One-Out Sensitivity Analyses for Total Sleep Time (TST), Time in Bed (TIB), Wake After Sleep Onset (WASO), and Sleep Efficiency (SE) in Patients With Clinical High Risk for Psychosis (CHR-P) and Schizophrenia Spectrum Disorders (SSD)

eFigure 31. Baujat Plots for Total Sleep Time (TST), Time in Bed (TIB), Wake After Sleep Onset (WASO), and Sleep Efficiency (SE) in Patients With Clinical High Risk for Psychosis (CHR-P) and Schizophrenia Spectrum Disorders (SSD)

eFigure 32. Funnel Plot and Egger Regression for the Primary Outcome (Total Sleep Time [TST] lnVR) in Patients With Schizophrenia Spectrum Disorders (SSD)

eFigure 33. Funnel Plots for Key Secondary Outcomes (Time in Bed [TIB], Wake After Sleep Onset [WASO], and Sleep Efficiency [SE]) in Patients With Clinical High Risk for Psychosis (CHR-P) and Schizophrenia Spectrum Disorders (SSD)

eReferences

This supplementary material has been provided by the authors to give readers additional information about their work.

© 2026 Aronica R et al. *JAMA Network Open*.

eMethods. Full Search Strategy and Database Syntax

The initial search ran from database inception to April 29, 2024, and an updated search covering 30 April 2024 to 25 April 2026 was performed on 25 April 2026 using identical syntax. The updated search identified 104 additional records that were screened against the inclusion criteria; none met the criteria and no additional studies were added to the review. No language, date or publication-status restrictions were applied.

PubMed

("actigraphy"[MeSH] OR "actigraphy"[All Fields] OR "actimetry"[All Fields] OR "wearable electronic devices"[MeSH] OR "wearable*"[All Fields] OR "accelerometer*"[All Fields] OR "wrist-worn"[All Fields]) AND ("schizophrenia"[MeSH] OR "schizo*"[All Fields] OR "psychosis"[All Fields] OR "psychotic disorder*"[All Fields] OR "clinical high risk"[All Fields] OR "ultra-high risk"[All Fields] OR "prodromal"[All Fields] OR "at-risk mental state*"[All Fields])

Embase / MEDLINE (Ovid)

1. exp actigraphy/ OR actigraph*.tw,kw. OR actimetry.tw,kw. OR accelerometer*.tw,kw. OR wearable*.tw,kw. OR wrist-worn.tw,kw.
2. exp schizophrenia/ OR schizo*.tw,kw. OR psychosis.tw,kw. OR psychotic*.tw,kw. OR "clinical high risk".tw,kw. OR "ultra-high risk".tw,kw. OR prodromal.tw,kw. OR "at-risk mental state*".tw,kw.
3. 1 AND 2

Cochrane CENTRAL

#1 MeSH descriptor: [Actigraphy] explode all trees
#2 (actigraph* OR actimetry OR accelerometer* OR wearable* OR "wrist-worn"):ti,ab,kw
#3 #1 OR #2
#4 MeSH descriptor: [Schizophrenia] explode all trees
#5 (schizo* OR psychosis OR psychotic* OR "clinical high risk" OR "ultra-high risk" OR prodromal OR "at-risk mental state*"):ti,ab,kw
#6 #4 OR #5
#7 #3 AND #6

Trial registries and grey literature

ClinicalTrials.gov, EU Clinical Trials Register, WHO ICTRP and OpenGrey were searched with the simplified syntax ("actigraphy" OR "wearable" OR "accelerometer") AND ("schizophrenia" OR "psychosis" OR "clinical high risk").

© 2026 Aronica R et al. *JAMA Network Open*.

eTable 1. ROBINS-E Ratings Per Study and Per Domain (Primary Outcome)

Study	D1	D2	D3	D4	D5	D6	D7	Overall
**Afonso2011** [1]	SC	L	SC	L	L	L	SC	SC
**Apiquian2008** [2]	SC	L	SC	H	L	L	SC	H
**Bartsh2021** [3]	SC	L	L	L	SC	L	SC	SC
**Henning2020** [4]	L	L	L	L	L	L	SC	SC
**Kammerer2021** [5]	L	L	SC	L	L	L	SC	SC
**Lunsford-Avery2015** [6]	SC	L	L	L	L	L	SC	SC
**Martin2005** [7]	L	L	SC	L	L	L	SC	SC
**Mayeli2023** [8]	L	L	SC	L	H	L	SC	SC
**Nordholm2023** [9]	L	L	L	L	L	L	SC	SC
**Ristanovic2022** [10]	SC	L	L	L	L	L	SC	SC
**Robillard2015** [11]	L	L	SC	L	L	L	SC	SC
**Skeldon2022** [12]	L	L	SC	L	L	L	SC	SC
**Skowerska2010** [13]	H	L	SC	L	SC	L	SC	H
**Tous-Espelosin2021** [14]	SC	L	SC	L	SC	L	SC	H
**Wamsley2012** [15]	SC	L	SC	L	SC	L	SC	H
**Waters2011** [16]	L	L	SC	L	L	L	SC	SC
**Wichniak2011** [17]	SC	L	SC	SC	L	L	SC	H
**Wulff2012** [18]	SC	L	SC	L	L	L	SC	SC

Domains: D1, confounding; D2, measurement of the exposure; D3, selection of participants; D4, post-exposure interventions; D5, missing data; D6, measurement of the outcome; D7, reported result. Ratings: L = low; SC = some concerns; H = high. Summary: 5 of 18 studies (28.8%) were rated overall high risk; 13 of 18 (72.2%) were rated some concerns; no study was rated low. Concerns were concentrated in D3 (selection of participants) and D7 (reported result).

© 2026 Aronica R et al. *JAMA Network Open*.

eTable 2. Characteristics of Included Studies

Part A. Core study characteristics (reproduced from the master data extraction file)

Study	Group	Continent	n clinical / HC	Age (mean), clinical / HC	% female, clinical / HC	Antipsychotic use, %	PANSS total (mean)
**Afonso2011** [1]	SSD	Europe	34 / 34	33.8 / 34.7	35% / 44%	100%	66.9
**Apiquian2008** [2]	SSD	North America	17 / 17	28.5 / 30.3	50% / 70%	—	—
**Bartsh2021** [3]	CHR-P	Europe	19 / 16	—	—	—	—
**Henning2020** [4]	CHR-P	Europe	41 / 41	21.1 / 21.3	60% / 46%	—	—
**Kammerer2021** [5]	SSD	Europe	67 / 39	38.0 / 37.9	43% / 46%	84%	33.9
**Lunsford-Avery2015** [6]	CHR-P	North America	36 / 31	18.7 / 17.9	47% / 48%	6%	—
**Martin2005** [7]	SSD	North America	28 / 28	58.3 / 57.3	50% / 50%	—	—
**Mayeli2023** [8]	SSD	Europe	122 / 108	41.2 / 41.5	38% / 39%	99%	—
**Nordholm2023** [9]	CHR-P	Europe	68 / 36	23.9 / 23.8	58% / 47%	33%	—
**Ristanovic2022** [10]	CHR-P	North America	38 / 36	18.9 / 18.3	40% / 54%	—	—
**Robillard2015** [11]	SSD	Australia	30 / 41	22.5 / 25.3	33% / 54%	70%	—
**Skeldon2022** [12]	SSD	Europe	20 / 21	38.8 / 37.5	50% / 38%	100%	—
**Skowerska2010** [13]	SSD	Europe	23 / 23	40.5 / 40.5	61% / 61%	100%	24.2
**Tous-Espelosin2021** [14]	SSD	Europe	113 / 30	41.6 / 40.0	17% / 60%	100%	—
**Wamsley2012** [15]	SSD	North America	21 / 17	34.0 / 36.0	19% / 18%	100%	28.0
**Waters2011** [16]	SSD	Australia	6 / 7	44.3 / 42.7	17% / 43%	100%	—
**Wichniak2011** [17]	SSD	Europe	73 / 36	29.7 / 30.1	39% / 48%	100%	41.8
**Wulff2012** [18]	SSD	Europe	20 / 21	38.8 / 37.5	25% / 38%	60%	—

© 2026 Aronica R et al. *JAMA Network Open*.

Part B. Actigraphy methodological characteristics

Study	Device (manufacturer / model)	Consumer device	Sampling frequency, Hz	Sampling mode	Epoch, s	Recording (max nights)
**Afonso2011** [1]	SOMNOwatch	No	NR	NR	1	7
**Apiquian2008** [2]	Actiwatch-16, Mini Mitter Company, Bend, OR	No	32	fixed	60	6
**Bartsh2021** [3]	MotionWatch 8, Cambridge Neurotechnology	No	NR	NR	NR	14
**Henning2020** [4]	Actiwatch 2 (Philips Respironics)	Yes	NR	NR	30	14
**Kammerer2021** [5]	NR	nan	NR	NR	15	6
**Lunsford-Avery2015** [6]	ActiSleep monitors (ActiGraph; Pensacola, FL)	No	NR	NR	60	5
**Martin2005** [7]	ActiGraph GT9X, Pensacola	No	NR	NR	NR	3
**Mayeli2023** [8]	Actillume wrist actigraph	No	NR	NR	60	7
**Nordholm2023** [9]	ActiGraph wGT3X-BT	No	30	Fixed	60	1
**Ristanovic2022** [10]	ActiSleep monitors (ActiGraph; Pensacola, FL)	No	NR	NR	60	5
**Robillard2015** [11]	Actiwatch-64/L/2, Philips Respironics	Yes	NR	NR	60	22
**Skeldon2022** [12]	Actiwatch-L (Cambridge Neurotechnology Ltd)	No	NR	NR	120	42
**Skowerska2010** [13]	NR	nan	NR	NR	NR	—
**Tous-Espelosin2021** [14]	ActiGraph GT3X+, Pensacola	No	NR	NR	60	8
**Wamsley2012** [15]	The Mini-Mitter Actiwatch	No	NR	NR	15	5
**Waters2011** [16]	Actiwatch 2 (Philips Respironics)	Yes	NR	NR	60	28
**Wichniak2011** [17]	Actiwatch AW4, Cambridge Neurotechnology Inc., UK	No	NR	NR	30	7
**Wulff2012** [18]	Actiwatch-L (Cambridge Neurotechnology Ltd)	No	32	Fixed	120	42

eTable 3. Sensitivity Meta-Analysis Excluding Studies at Overall High Risk of Bias

© 2026 Aronica R et al. *JAMA Network Open*.

Outcome	Group	Metric	k all → excl	VR all (95% CI)	VR excl (95% CI)	I² excl, %	P excl	Direction
**TST**	CHR-P	lnVR	5 → 5	1.17 (0.95 to 1.45)	1.17 (0.95 to 1.45)	47	0.14	Direction preserved (not significant)
**TST**	CHR-P	lnCVR	5 → 5	1.18 (0.97 to 1.44)	1.18 (0.97 to 1.44)	36	0.10	Direction preserved (not significant)
**TST**	SSD	lnVR	13 → 8	1.58 (1.32 to 1.89)	1.71 (1.33 to 2.20)	76	<.001	Direction preserved (significant)
**TST**	SSD	lnCVR	13 → 8	1.25 (1.03 to 1.52)	1.36 (1.01 to 1.82)	81	0.04	Direction preserved (significant)
**TIB**	CHR-P	lnVR	2 → 2	1.34 (1.04 to 1.72)	1.34 (1.04 to 1.72)	0	0.02	Direction preserved (significant)
**TIB**	CHR-P	lnCVR	2 → 2	1.30 (1.00 to 1.67)	1.30 (1.00 to 1.67)	0	0.05	Direction preserved (significant)
**TIB**	SSD	lnVR	4 → —	1.46 (1.21 to 1.75)	—	—	—	Not estimable after RoB exclusion
**TIB**	SSD	lnCVR	4 → —	1.15 (0.96 to 1.39)	—	—	—	Not estimable after RoB exclusion
**WASO**	CHR-P	lnVR	4 → 4	1.57 (1.07 to 2.30)	1.57 (1.07 to 2.30)	83	0.02	Direction preserved (significant)
**WASO**	CHR-P	lnCVR	4 → 4	1.30 (0.98 to 1.74)	1.30 (0.98 to 1.74)	48	0.07	Direction preserved (not significant)
**WASO**	SSD	lnVR	5 → 4	1.69 (1.16 to 2.44)	1.60 (1.01 to 2.53)	90	0.04	Direction preserved (significant)
**WASO**	SSD	lnCVR	5 → 4	1.20 (1.04 to 1.39)	1.16 (0.98 to 1.36)	0	0.08	Lost significance
**SE**	CHR-P	lnVR	5 → 5	1.42 (1.06 to 1.89)	1.42 (1.06 to 1.89)	72	0.02	Direction preserved (significant)
**SE**	CHR-P	lnCVR	5 → 5	1.45 (1.08 to 1.96)	1.45 (1.08 to 1.96)	73	0.01	Direction preserved (significant)
**SE**	SSD	lnVR	9 → 5	1.31 (1.04 to 1.64)	1.55 (1.09 to 2.20)	74	0.01	Direction preserved (significant)
**SE**	SSD	lnCVR	9 → 5	1.31 (1.03 to 1.67)	1.56 (1.08 to 2.26)	77	0.02	Direction preserved (significant)

Random-effects (REML) re-pooling of lnVR and lnCVR after excluding the 5 studies rated overall high risk of bias (Apiquian 2008, Skowerska 2010, Tous-Espelosín 2021, Wamsley 2012, Wichniak 2011). "Not estimable" indicates fewer than 2 studies remained after exclusion. The Direction column flags whether the post-exclusion estimate preserves the direction and statistical significance of the primary all-studies pool.

© 2026 Aronica R et al. *JAMA Network Open*.

eTable 4. Leave-One-Out Sensitivity Summary for the Primary or Significant Outcomes (lnVR Only)

**Outcome × Group**	**k**	**Pooled VR (95% CI)**	**Pooled I^2**	**Leave-one-out VR range**	**Studies whose omission impacts significance**
TST CHR-P	5	1.17 (0.95-1.45)	47%	1.05 – 1.24	None
TST SSD	13	1.58 (1.32-1.89)	70%	1.52 – 1.66	None
TIB CHR-P	2	1.34 (1.04-1.72)	0%	Not informative (k<3)	—
TIB SSD	4	1.46 (1.21-1.75)	0%	1.38 – 1.53	None
WASO CHR-P	4	1.57 (1.07-2.30)	83%	1.40 – 1.80	Lunsford-Avery2015, Ristanovic2022
WASO SSD	5	1.69 (1.16-2.44)	88%	1.44 – 1.85	None
SE CHR-P	5	1.42 (1.06-1.89)	72%	1.28 – 1.53	Lunsford-Avery2015, Ristanovic2022
SE SSD	9	1.31 (1.04-1.64)	71%	1.22 – 1.38	Afonso2011, Robillard2015, Tous-Espelosin2021, Waters2011

Random-effects (REML) leave-one-out sensitivity diagnostics for the six primary or significant outcomes (lnVR only). For each outcome × group, k studies were re-pooled k times (each time omitting one study); "Leave-one-out VR range" is the minimum-to-maximum back-transformed VR across those re-pools, and the rightmost column lists the studies whose omission changes the binary statistical-significance status of the pooled estimate (95% CI excluding vs including 1). Per-study leave-one-out forest plots are shown in eFigure 30 and the corresponding Baujat plots in eFigure 31.

© 2026 Aronica R et al. *JAMA Network Open*.

eTable 5. Subgroup Analyses Stratified by Sampling Epoch

Outcome	Group	Metric	Stratifier	Stratum	k	VR (95% CI)	I², %	τ²	P (between)
TST	CHR-P	lnVR	Sampling epoch	≤30 s	1	1.01 (0.74 to 1.38)	—	—	—
TST	CHR-P	lnVR	Sampling epoch	≥60 s	3	1.24 (0.91 to 1.70)	65	0.050	—
TST	CHR-P	lnVR	Device class	Consumer-grade	1	1.01 (0.74 to 1.38)	—	—	—
TST	CHR-P	lnVR	Device class	Medical-grade	4	1.22 (0.95 to 1.57)	51	0.033	—
TST	CHR-P	lnCVR	Sampling epoch	≤30 s	1	0.99 (0.72 to 1.36)	—	—	—
TST	CHR-P	lnCVR	Sampling epoch	≥60 s	3	1.26 (0.96 to 1.65)	50	0.029	—
TST	CHR-P	lnCVR	Device class	Consumer-grade	1	0.99 (0.72 to 1.36)	—	—	—
TST	CHR-P	lnCVR	Device class	Medical-grade	4	1.24 (1.00 to 1.55)	34	0.017	—
TST	SSD	lnVR	Sampling epoch	≤30 s	4	1.63 (1.27 to 2.10)	56	0.037	0.69
TST	SSD	lnVR	Sampling epoch	≥60 s	7	1.49 (1.11 to 2.02)	79	0.118	0.69
TST	SSD	lnVR	Device class	Consumer-grade	2	1.79 (1.31 to 2.45)	0	0.000	0.71
TST	SSD	lnVR	Device class	Medical-grade	9	1.58 (1.23 to 2.04)	80	0.117	0.71
TST	SSD	lnCVR	Sampling epoch	≤30 s	4	1.28 (0.99 to 1.65)	55	0.037	0.69
TST	SSD	lnCVR	Sampling epoch	≥60 s	7	1.16 (0.84 to 1.60)	80	0.139	0.69
TST	SSD	lnCVR	Device class	Consumer-grade	2	1.59 (1.15 to 2.19)	0	0.000	0.49
TST	SSD	lnCVR	Device class	Medical-grade	9	1.22 (0.93 to 1.61)	82	0.139	0.49
TIB	CHR-P	lnVR	Sampling epoch	≥60 s	1	1.42 (1.07 to 1.90)	—	—	—
TIB	CHR-P	lnVR	Device class	Medical-grade	2	1.34 (1.04 to 1.72)	0	0.000	—
TIB	CHR-P	lnCVR	Sampling epoch	≥60 s	1	1.36 (1.01 to 1.83)	—	—	—
TIB	CHR-P	lnCVR	Device class	Medical-grade	2	1.30 (1.00 to 1.67)	0	0.000	—
TIB	SSD	lnVR	Sampling epoch	≤30 s	2	1.34 (1.05 to 1.71)	0	0.000	—
TIB	SSD	lnVR	Device class	Medical-grade	3	1.45 (1.18 to 1.77)	0	0.000	—

© 2026 Aronica R et al. *JAMA Network Open*.

TIB	SSD	lnCVR	Sampling epoch	≤30 s	2	1.03 (0.80 to 1.32)	0	0.000	—
TIB	SSD	lnCVR	Device class	Medical-grade	3	1.13 (0.92 to 1.39)	0	0.000	—
WASO	CHR-P	lnVR	Sampling epoch	≤30 s	1	1.20 (0.88 to 1.64)	—	—	—
WASO	CHR-P	lnVR	Sampling epoch	≥60 s	3	1.72 (1.06 to 2.79)	85	0.156	—
WASO	CHR-P	lnVR	Device class	Consumer-grade	1	1.20 (0.88 to 1.64)	—	—	—
WASO	CHR-P	lnVR	Device class	Medical-grade	3	1.72 (1.06 to 2.79)	85	0.156	—
WASO	CHR-P	lnCVR	Sampling epoch	≤30 s	1	1.09 (0.75 to 1.58)	—	—	—
WASO	CHR-P	lnCVR	Sampling epoch	≥60 s	3	1.39 (0.96 to 2.02)	55	0.059	—
WASO	CHR-P	lnCVR	Device class	Consumer-grade	1	1.09 (0.75 to 1.58)	—	—	—
WASO	CHR-P	lnCVR	Device class	Medical-grade	3	1.39 (0.96 to 2.02)	55	0.059	—
WASO	SSD	lnVR	Sampling epoch	≤30 s	1	1.19 (0.89 to 1.57)	—	—	—
WASO	SSD	lnVR	Sampling epoch	≥60 s	3	1.53 (1.12 to 2.11)	77	0.060	—
WASO	SSD	lnVR	Device class	Consumer-grade	1	1.42 (1.01 to 1.99)	—	—	—
WASO	SSD	lnVR	Device class	Medical-grade	3	2.02 (1.16 to 3.53)	92	0.221	—
WASO	SSD	lnCVR	Sampling epoch	≤30 s	1	1.02 (0.65 to 1.60)	—	—	—
WASO	SSD	lnCVR	Sampling epoch	≥60 s	3	1.26 (1.07 to 1.48)	0	0.000	—
WASO	SSD	lnCVR	Device class	Consumer-grade	1	1.10 (0.74 to 1.64)	—	—	—
WASO	SSD	lnCVR	Device class	Medical-grade	3	1.25 (1.06 to 1.48)	0	0.000	—
SE	CHR-P	lnVR	Sampling epoch	≤30 s	1	1.11 (0.81 to 1.51)	—	—	—
SE	CHR-P	lnVR	Sampling epoch	≥60 s	3	1.61 (1.07 to 2.44)	80	0.107	—
SE	CHR-P	lnVR	Device class	Consumer-grade	1	1.11 (0.81 to 1.51)	—	—	—
SE	CHR-P	lnVR	Device class	Medical-grade	4	1.51 (1.08 to 2.12)	72	0.084	—
SE	CHR-P	lnCVR	Sampling epoch	≤30 s	1	1.12 (0.82 to 1.53)	—	—	—
SE	CHR-P	lnCVR	Sampling epoch	≥60 s	3	1.66 (1.09 to 2.55)	81	0.115	—

© 2026 Aronica R et al. *JAMA Network Open*.

SE	CHR-P	lnCVR	Device class	Consumer-grade	1	1.12 (0.82 to 1.53)	—	—	—
SE	CHR-P	lnCVR	Device class	Medical-grade	4	1.56 (1.10 to 2.20)	74	0.091	—
SE	SSD	lnVR	Sampling epoch	≤30 s	4	1.13 (0.77 to 1.65)	81	0.122	0.11
SE	SSD	lnVR	Sampling epoch	≥60 s	4	1.58 (1.30 to 1.91)	0	0.000	0.11
SE	SSD	lnVR	Device class	Consumer-grade	2	2.05 (1.07 to 3.93)	56	0.138	0.19
SE	SSD	lnVR	Device class	Medical-grade	5	1.29 (0.95 to 1.76)	73	0.088	0.19
SE	SSD	lnCVR	Sampling epoch	≤30 s	4	1.12 (0.75 to 1.70)	83	0.144	0.12
SE	SSD	lnCVR	Sampling epoch	≥60 s	4	1.59 (1.31 to 1.93)	0	0.000	0.12
SE	SSD	lnCVR	Device class	Consumer-grade	2	2.04 (1.12 to 3.71)	49	0.106	0.19
SE	SSD	lnCVR	Device class	Medical-grade	5	1.30 (0.94 to 1.79)	76	0.100	0.19

Subgroup random-effects (REML) meta-analyses of the natural log of the variability ratio (lnVR) and the natural log of the coefficient of variation ratio (lnCVR) for each primary or significant outcome × diagnostic group, stratified by actigraphy sampling epoch (≤30 s vs ≥60 s) and by device class (medical-grade vs consumer-grade). The between-subgroup P-value is the omnibus moderator Q from a meta-regression on the stratifier; it is reported only when both strata contain ≥2 contributing studies. Single-study strata (k = 1) are reported descriptively with a Wald 95% CI computed from the per-study lnVR / lnCVR and its standard error.

eTable 6. Small-Study Effects/Publication-Bias Assessment Across the Primary and Significant Outcomes

**Outcome × Group**	**k**	**Egger applicable?**	**Egger intercept (95% CI)**	**P**	**Funnel-plot assessment**
**SSD TST**	13	Yes (k ≥ 10)	0.49 (-0.06 to 1.05)	0.89	No statistically detectable funnel-plot asymmetry; visual inspection consistent with symmetry (eFigure 32)
**TIB CHR-P**	2	No (k < 10)	—	—	Visual only; too few studies for meaningful funnel inspection (eFigure 33)
**TIB SSD**	4	No (k < 10)	—	—	Visual only; no obvious asymmetry (eFigure 33)
**WASO CHR-P**	4	No (k < 10)	—	—	Visual only; no obvious asymmetry (eFigure 33)
**WASO SSD**	5	No (k < 10)	—	—	Visual only; no obvious asymmetry (eFigure 33)
**SE CHR-P**	5	No (k < 10)	—	—	Visual only; no obvious asymmetry (eFigure 33)
**SE SSD**	9	No (k < 10)	—	—	Visual only; no obvious asymmetry (eFigure 33)

Egger's regression for funnel-plot asymmetry was applied only to outcomes with ≥10 contributing studies, as recommended by the Cochrane Handbook (the test is underpowered with smaller samples). Of the 6 primary or significant outcomes, none reached k = 10; only the primary outcome SSD TST lnVR (k = 13) did. For the other 6 outcomes, funnel plots were generated for visual inspection only (eFigure 33). The funnel plot and Egger scatter for SSD TST are shown in eFigure 32.

© 2026 Aronica R et al. *JAMA Network Open*.

eFigure 1. Total Sleep Time (TST)—Patients With Clinical High Risk for Psychosis (CHR-P): Natural Logarithm of the Variability Ratio (lnVR) (*k* = 5; Restricted Maximum Likelihood [REML])

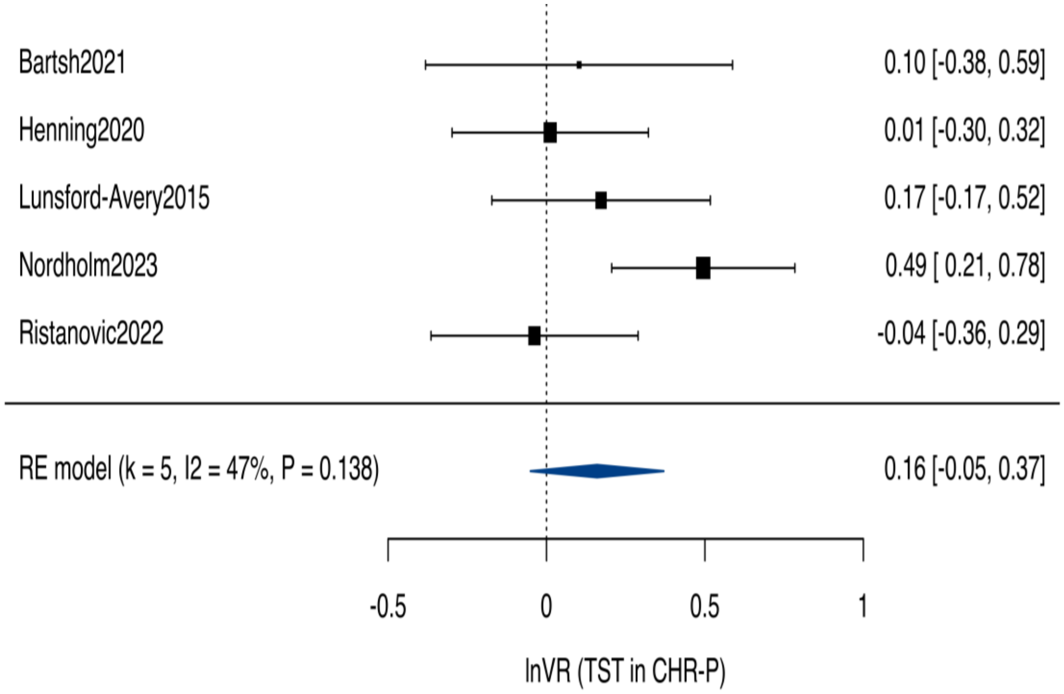

© 2026 Aronica R et al. *JAMA Network Open*.

eFigure 2. Total Sleep Time—CHR-P: lnCVR (*k* = 5; REML)

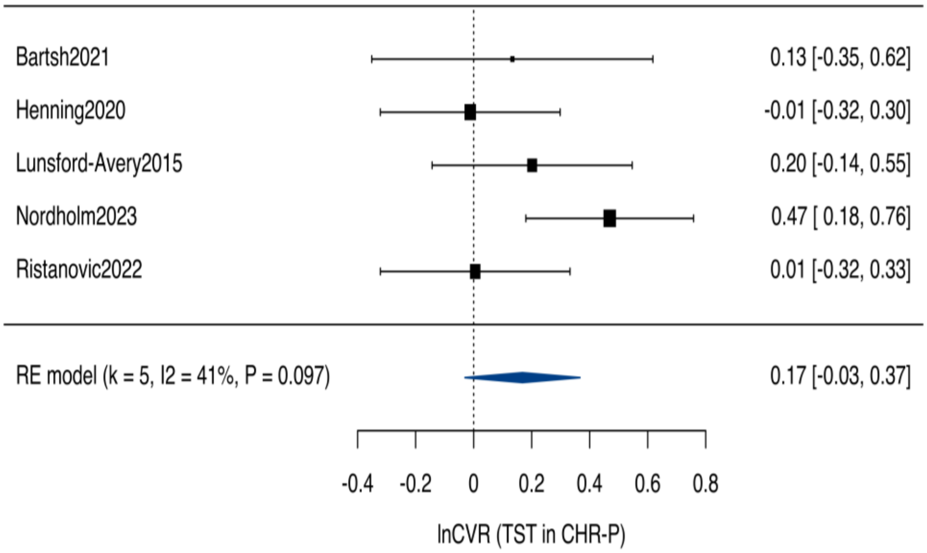

© 2026 Aronica R et al. *JAMA Network Open*.

eFigure 3. Total Sleep Time—CHR-P: Meta-Regression by Antipsychotic Use

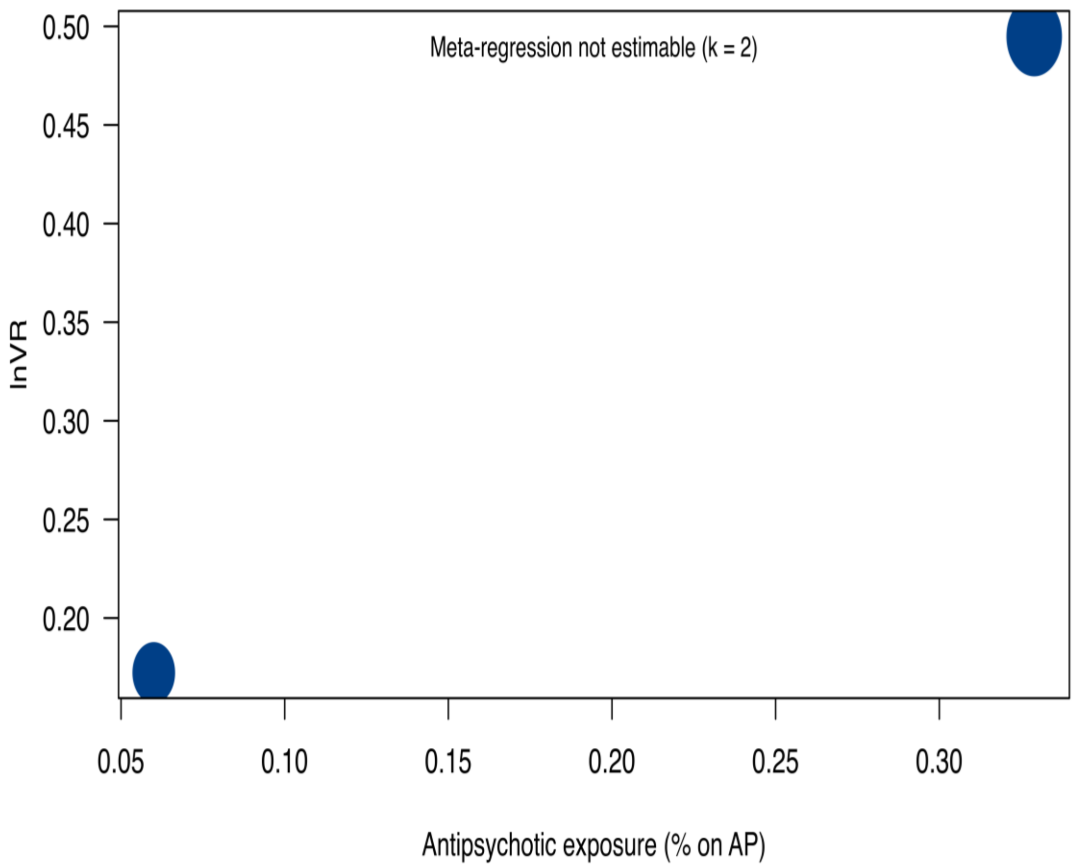

© 2026 Aronica R et al. *JAMA Network Open*.

eFigure 4. Total Sleep Time—CHR-P: Meta-Regression by Sex

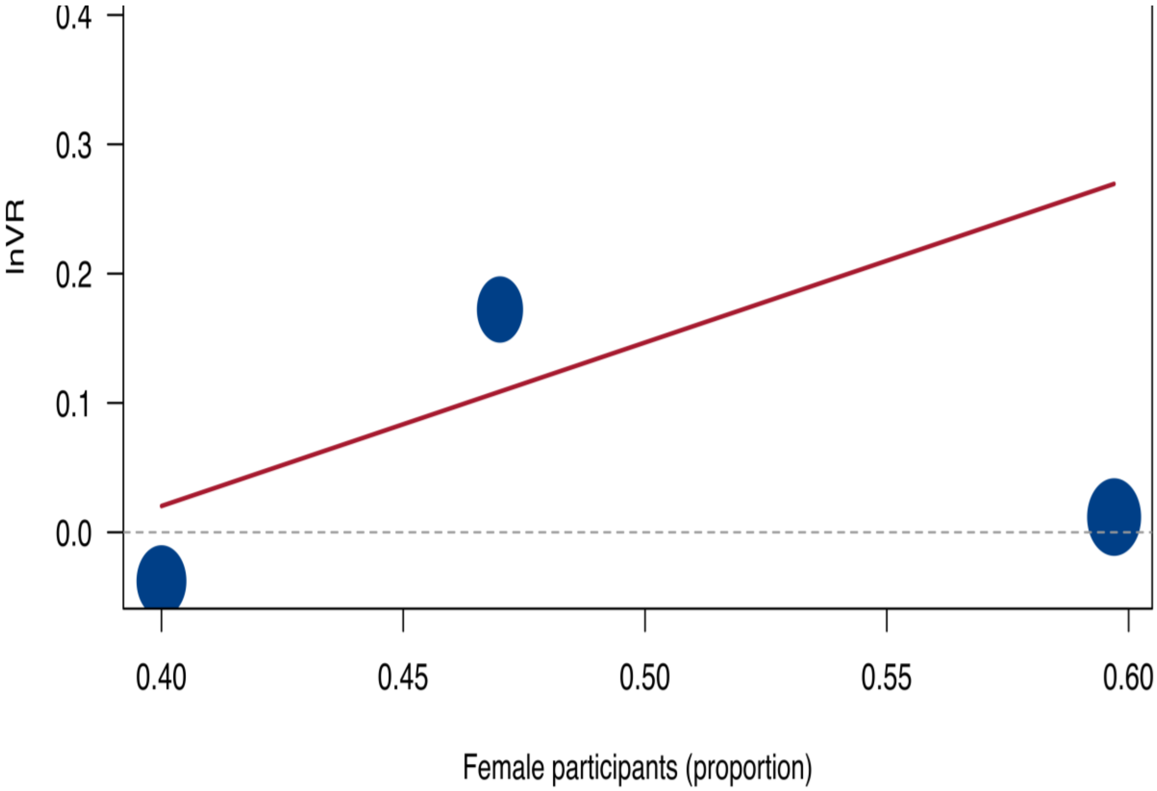

© 2026 Aronica R et al. *JAMA Network Open*.

eFigure 5. Total Sleep Time—CHR-P: Meta-Regression by Age

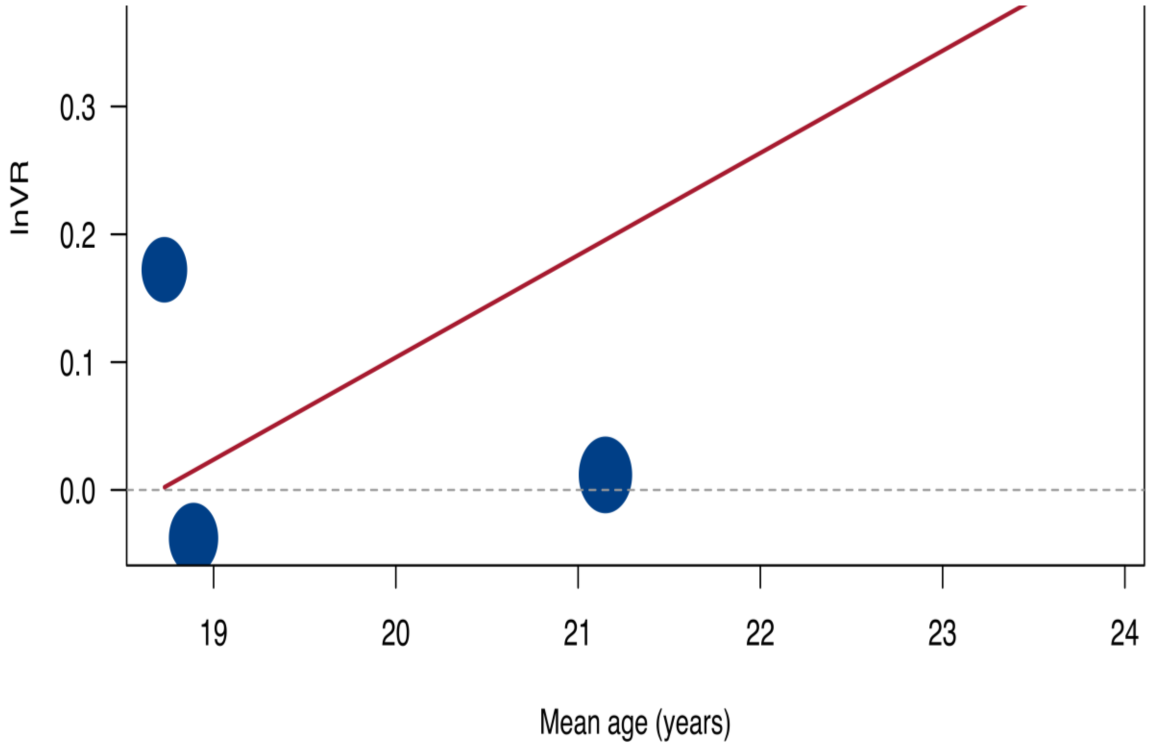

© 2026 Aronica R et al. *JAMA Network Open*.

eFigure 6. Total Sleep Time—Patients With Schizophrenia Spectrum Disorders (SSD): lnVR (*k* = 13; REML)

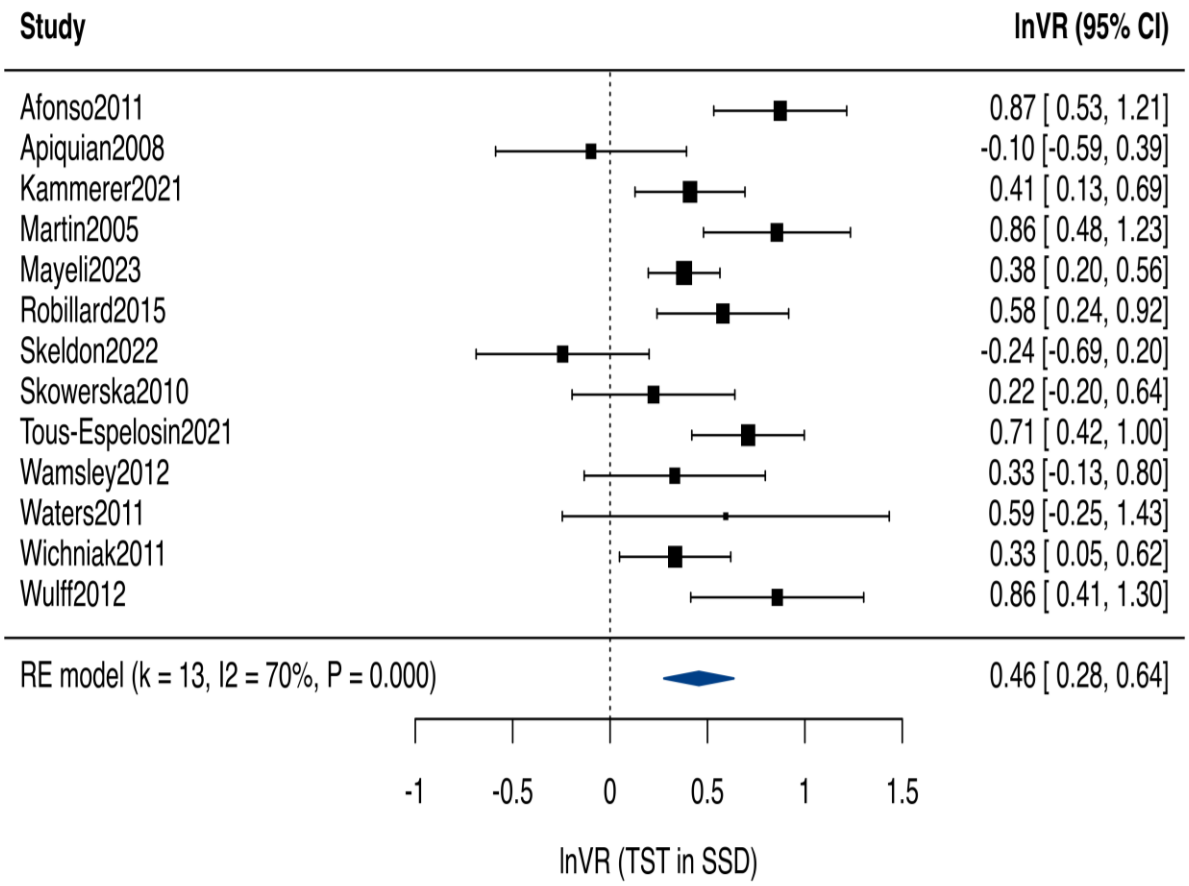

© 2026 Aronica R et al. *JAMA Network Open*.

eFigure 7. Total Sleep Time—SSD: lnCVR (*k* = 13; REML)

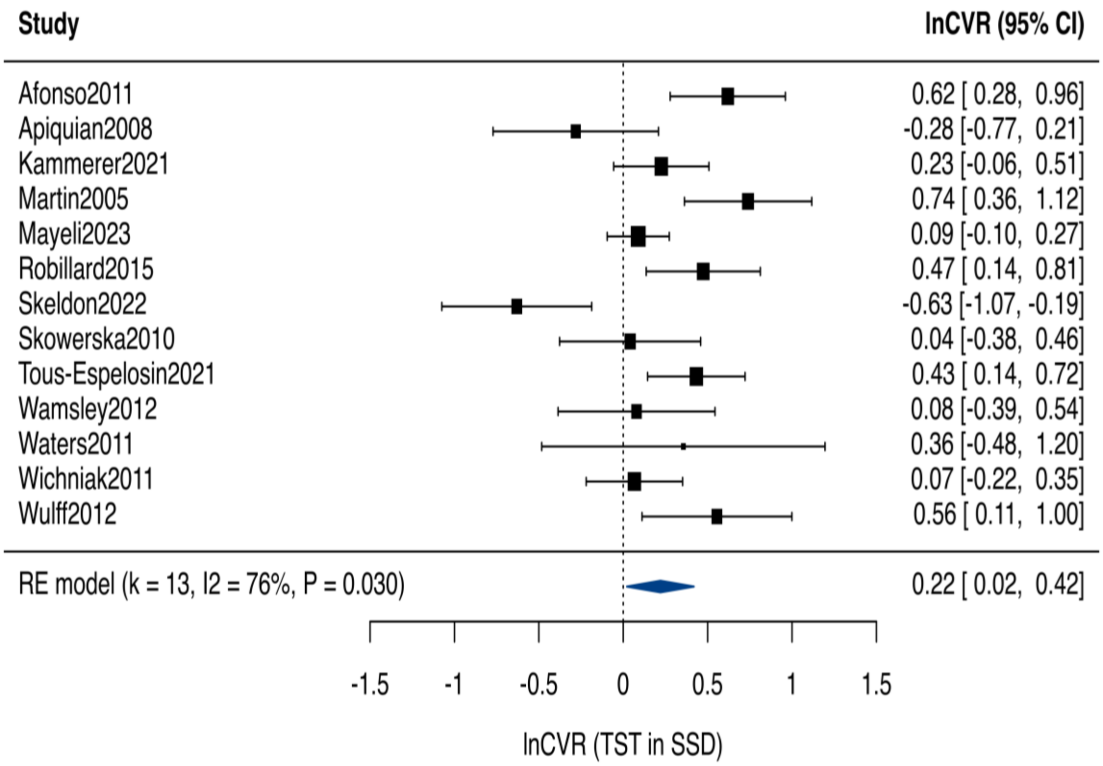

© 2026 Aronica R et al. *JAMA Network Open*.

eFigure 8. Total Sleep Time—SSD: Meta-Regression by Antipsychotic Use

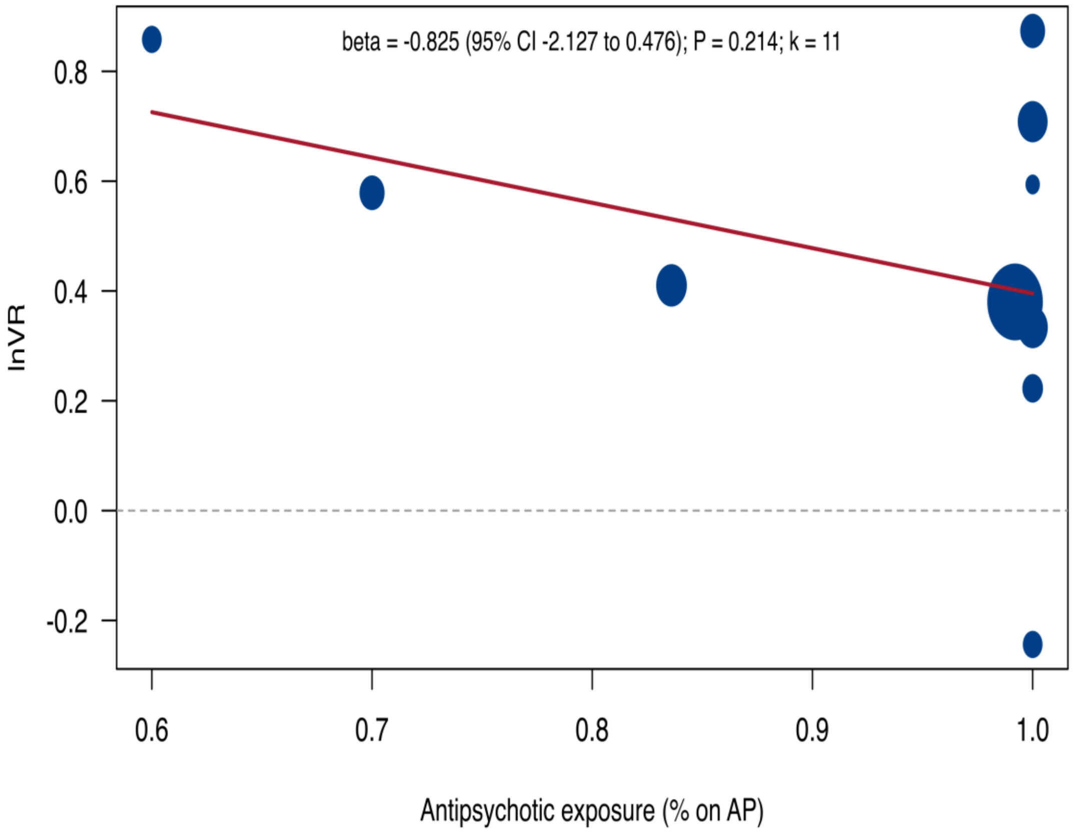

© 2026 Aronica R et al. *JAMA Network Open*.

eFigure 9. Total Sleep Time—SSD: Meta-Regression by Sex

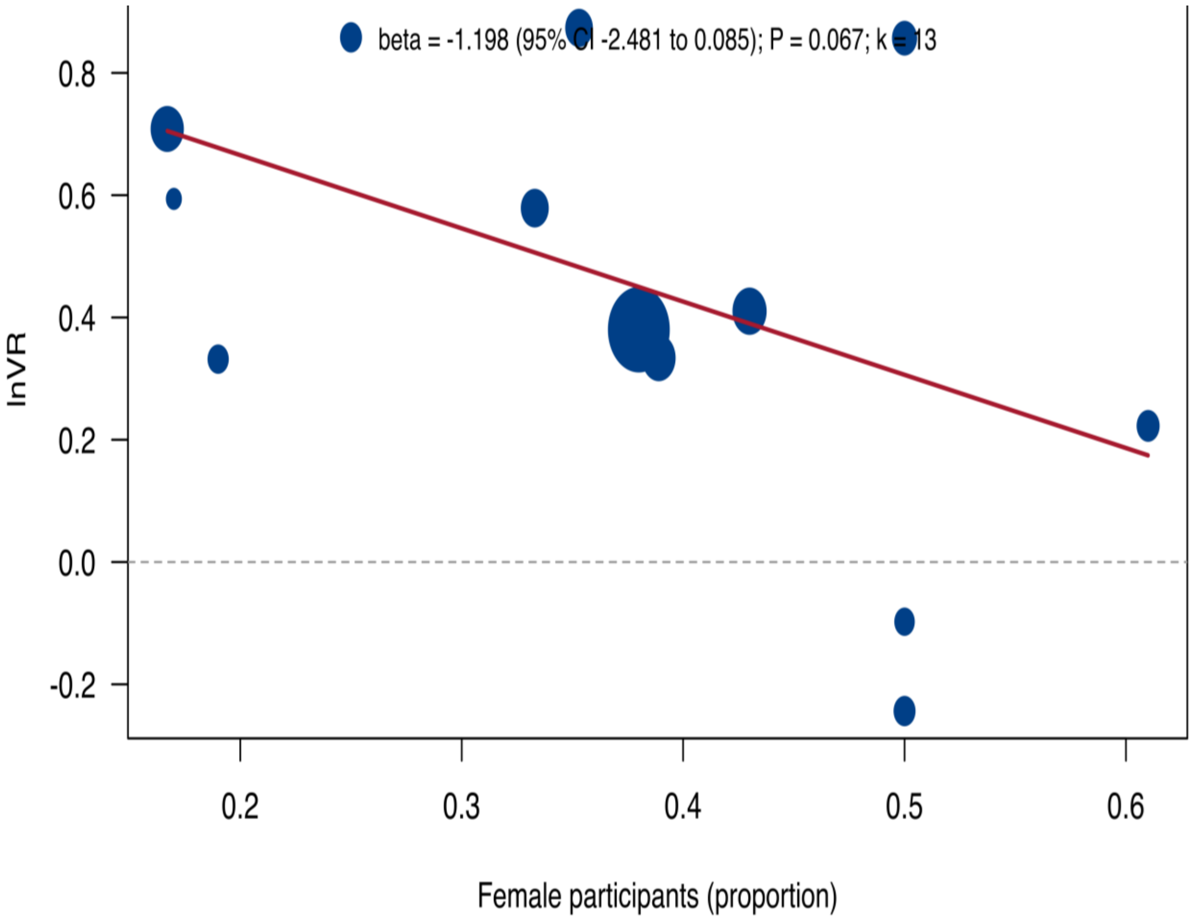

© 2026 Aronica R et al. *JAMA Network Open*.

eFigure 10. Total Sleep Time—SSD: Meta-Regression by Age

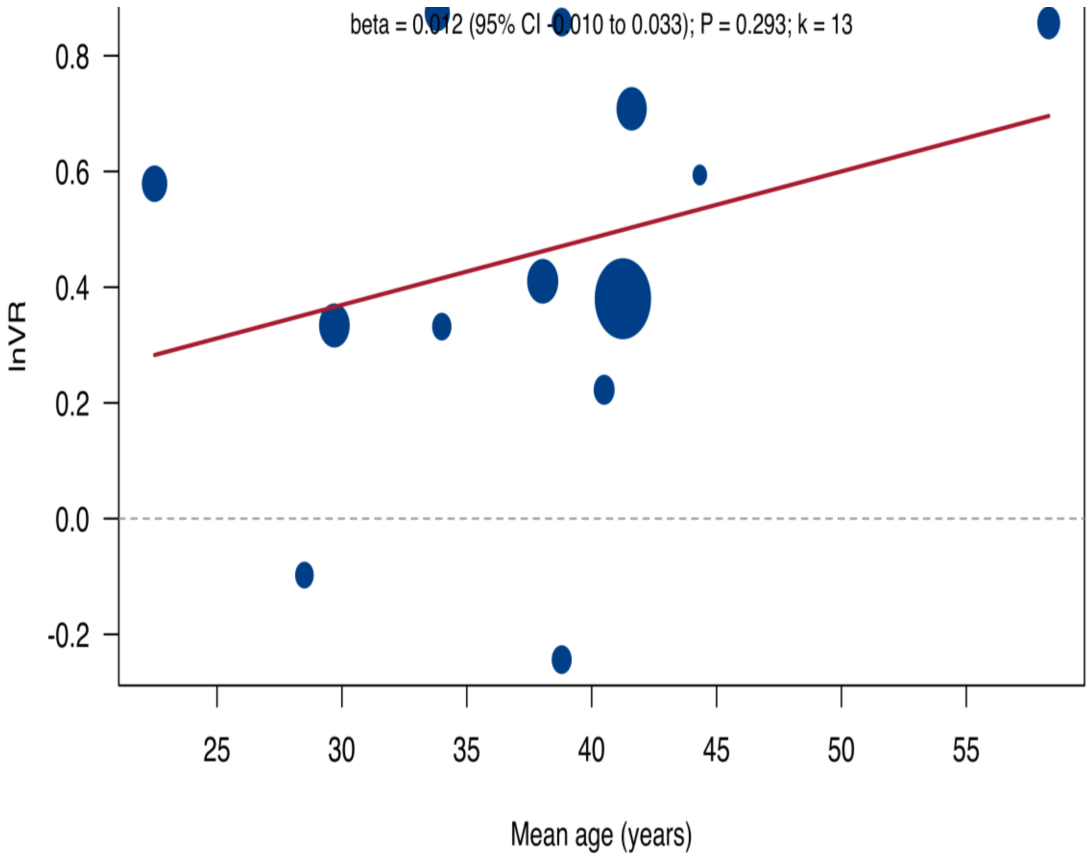

© 2026 Aronica R et al. *JAMA Network Open*.

eFigure 11. Time in Bed (TIB)—CHR-P: lnVR (k = 2; REML)

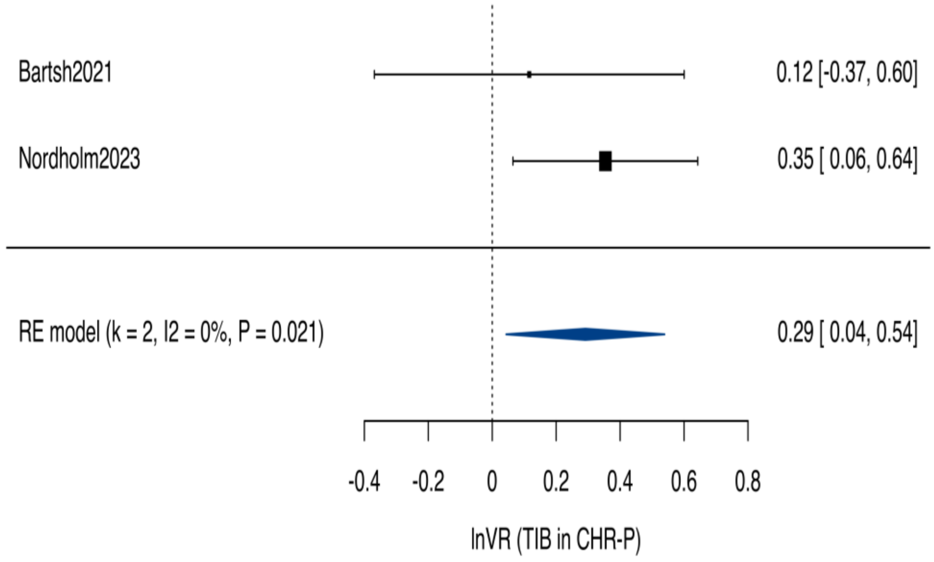

© 2026 Aronica R et al. *JAMA Network Open*.

eFigure 12. Time in Bed—CHR-P: lnCVR (*k* = 2; REML)

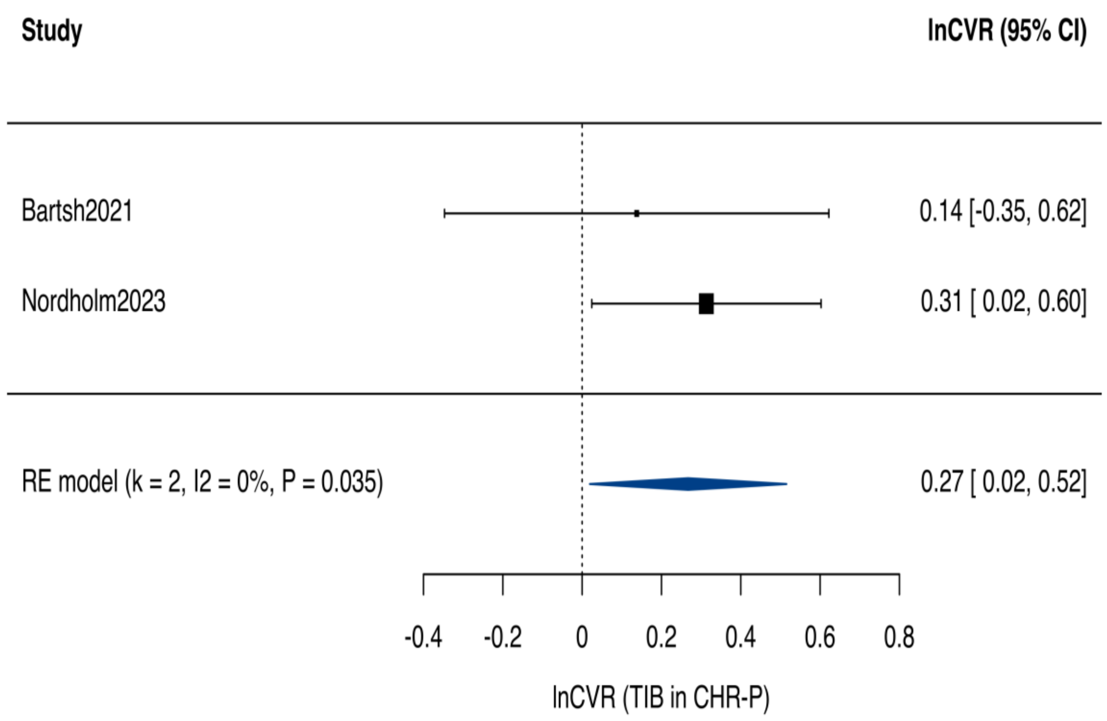

© 2026 Aronica R et al. *JAMA Network Open*.

eFigure 13. Time in Bed—SSD: lnVR (*k* = 4; REML)

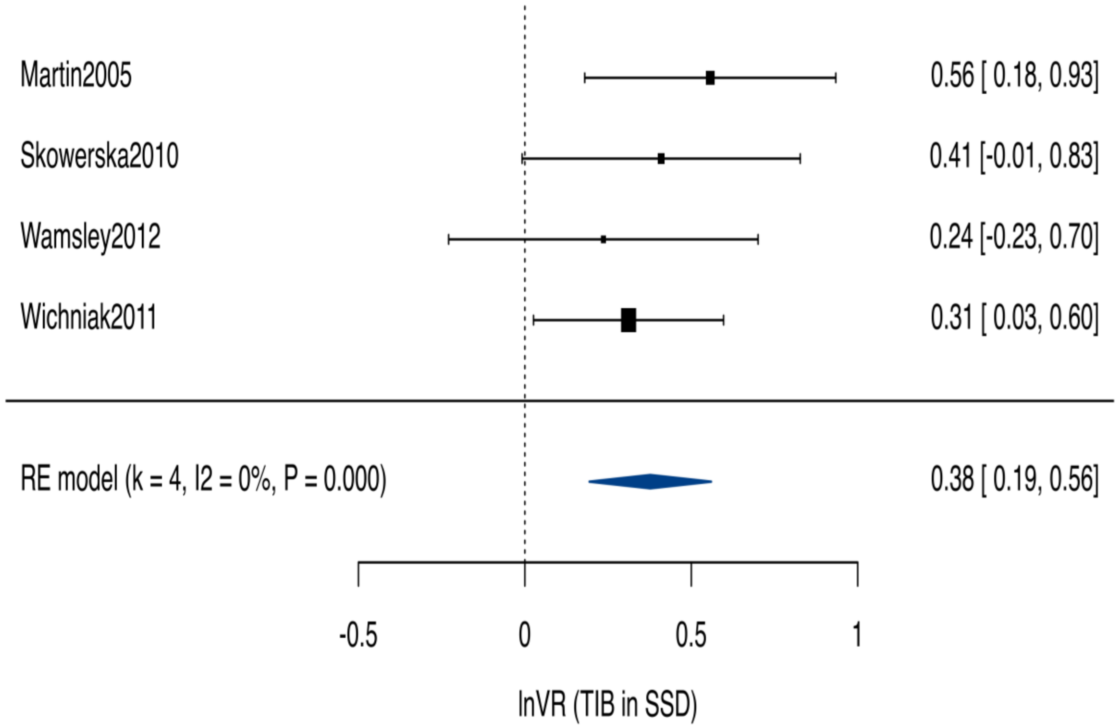

© 2026 Aronica R et al. *JAMA Network Open*.

eFigure 14. Time in Bed—SSD: lnCVR (*k* = 4; REML)

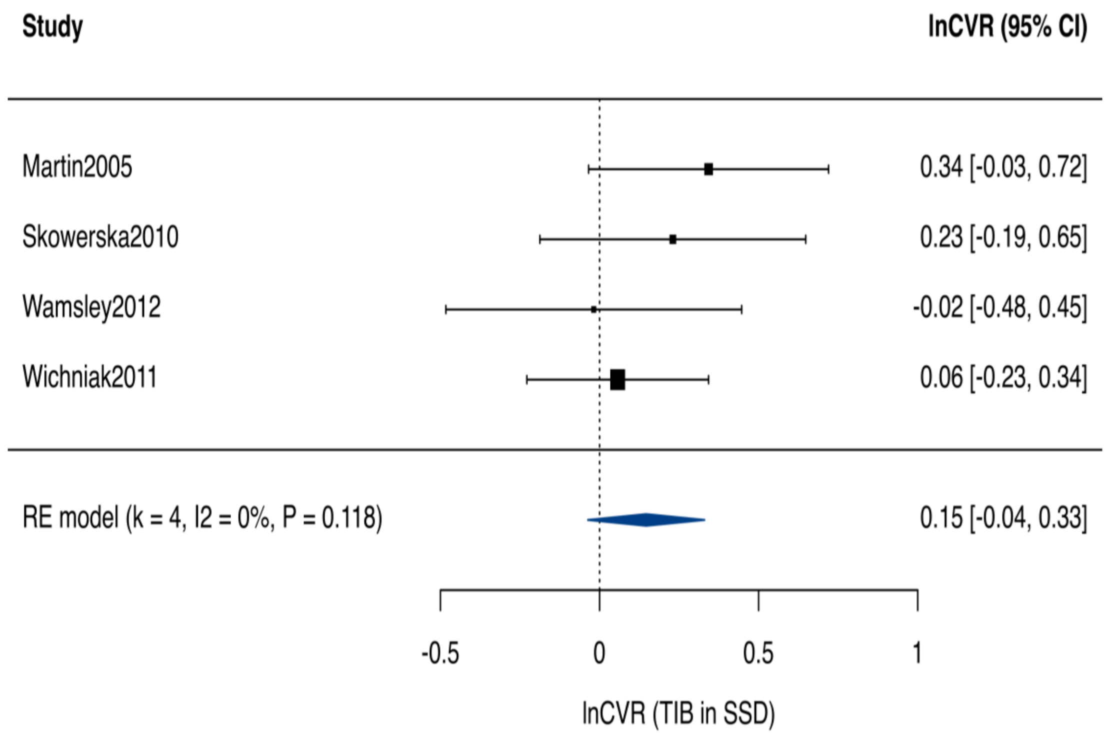

© 2026 Aronica R et al. *JAMA Network Open*.

eFigure 15. Sleep Latency (SL)—CHR-P: lnVR (*k* = 1)

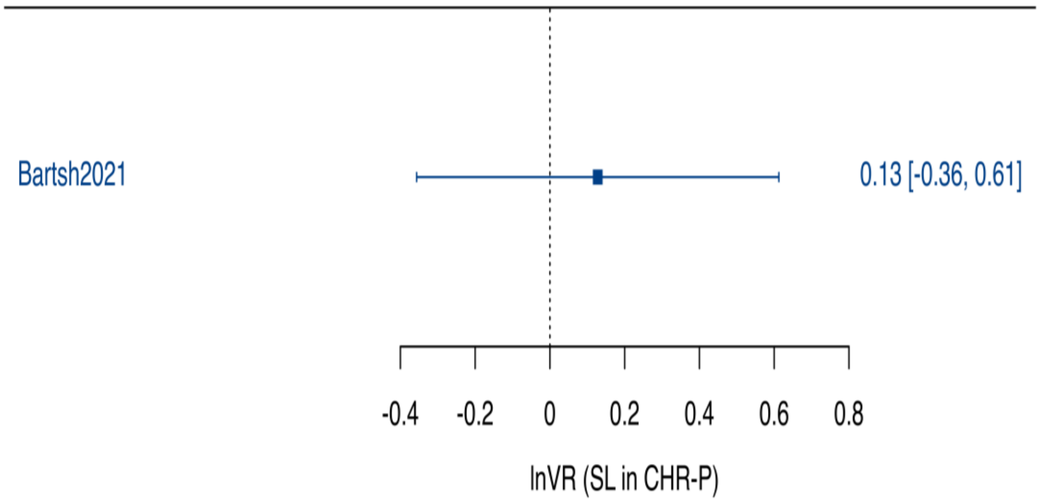

© 2026 Aronica R et al. *JAMA Network Open*.

eFigure 16. Sleep Latency—CHR-P: lnCVR (*k* = 1)

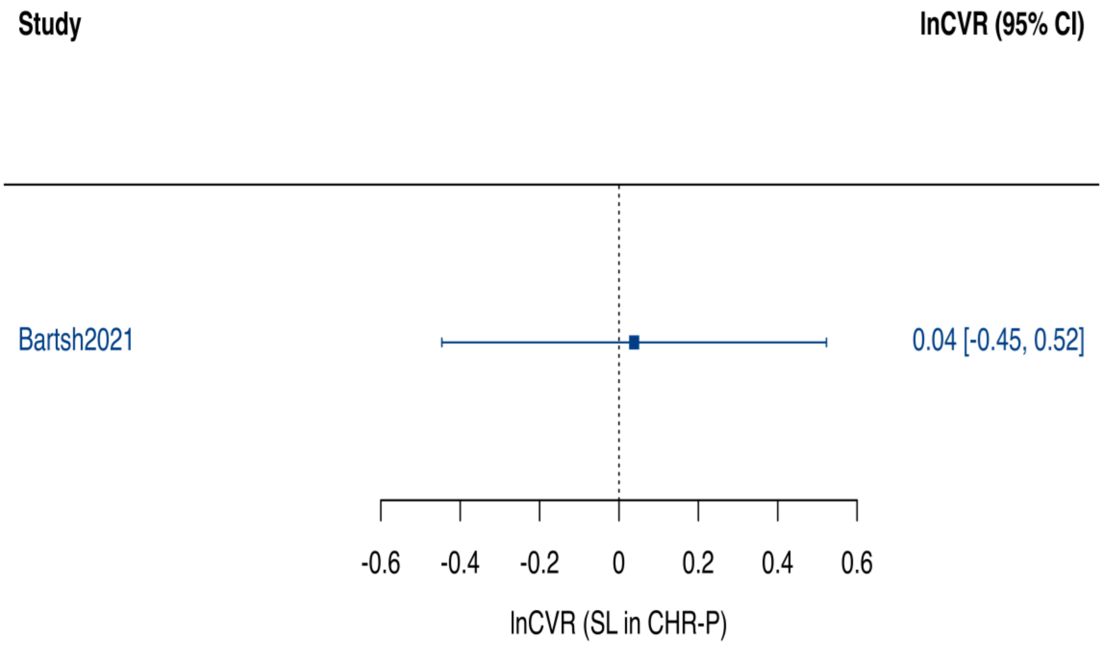

© 2026 Aronica R et al. *JAMA Network Open*.

eFigure 17. Sleep Latency—SSD: lnVR (*k* = 6; REML)

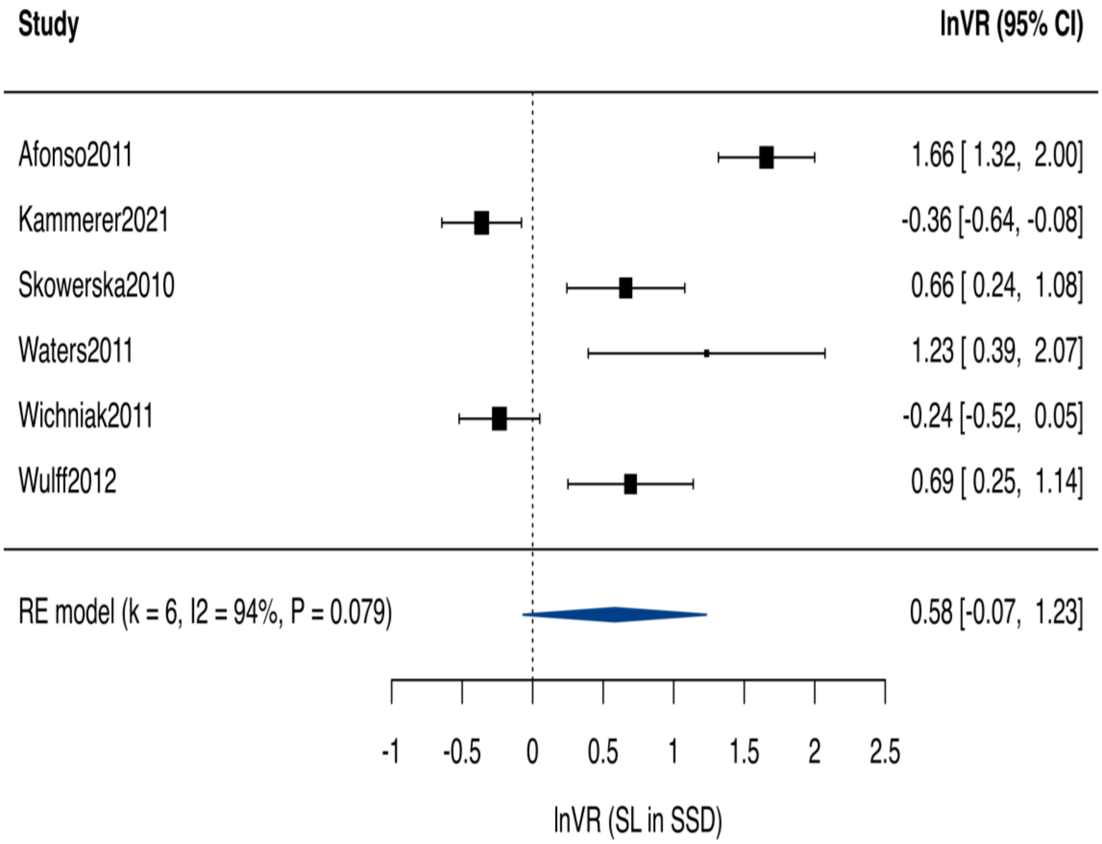

© 2026 Aronica R et al. *JAMA Network Open*.

eFigure 18. Sleep Latency—SSD: lnCVR (*k* = 6; REML)

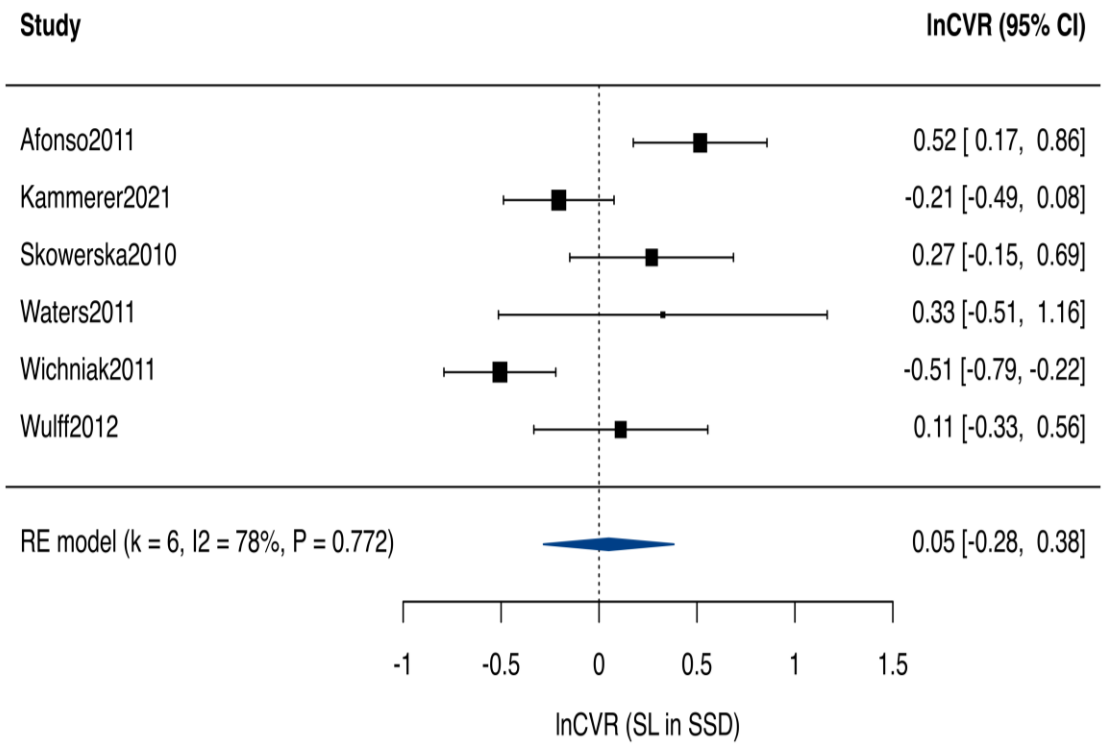

© 2026 Aronica R et al. *JAMA Network Open*.

eFigure 19. Number of Awakenings (AWK)—CHR-P: lnVR (*k* = 2; REML)

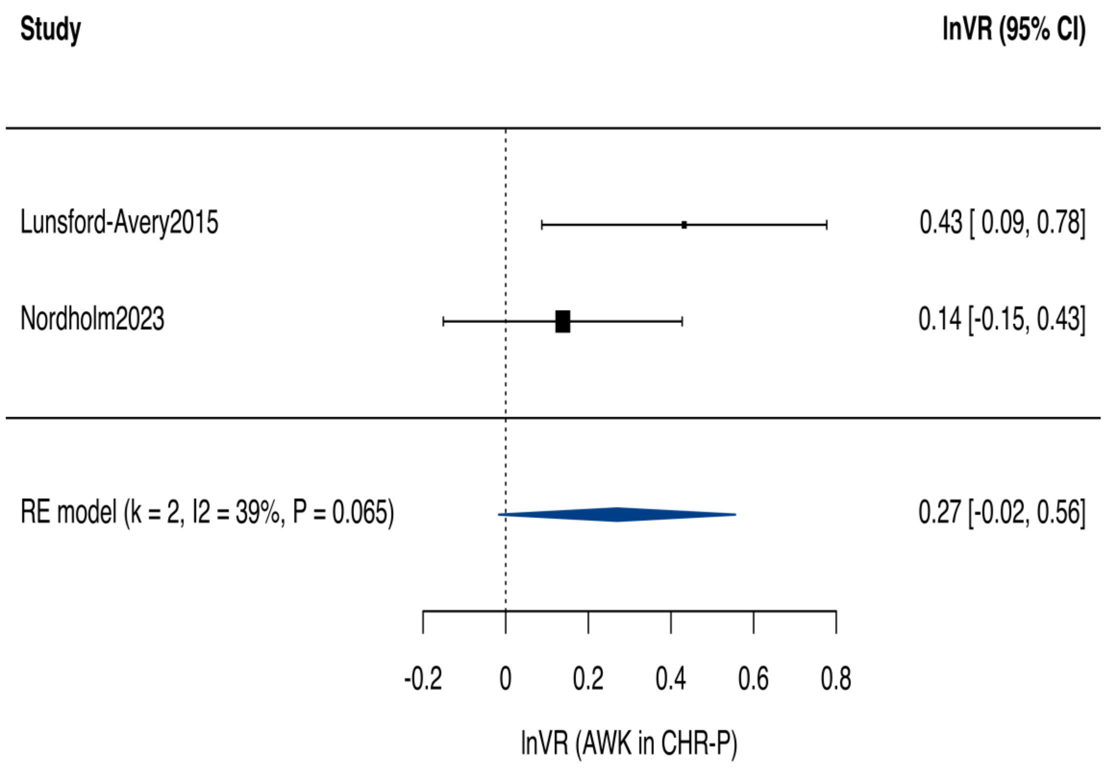

© 2026 Aronica R et al. *JAMA Network Open*.

eFigure 20. Number of Awakenings—CHR-P: lnCVR (*k* = 2; REML)

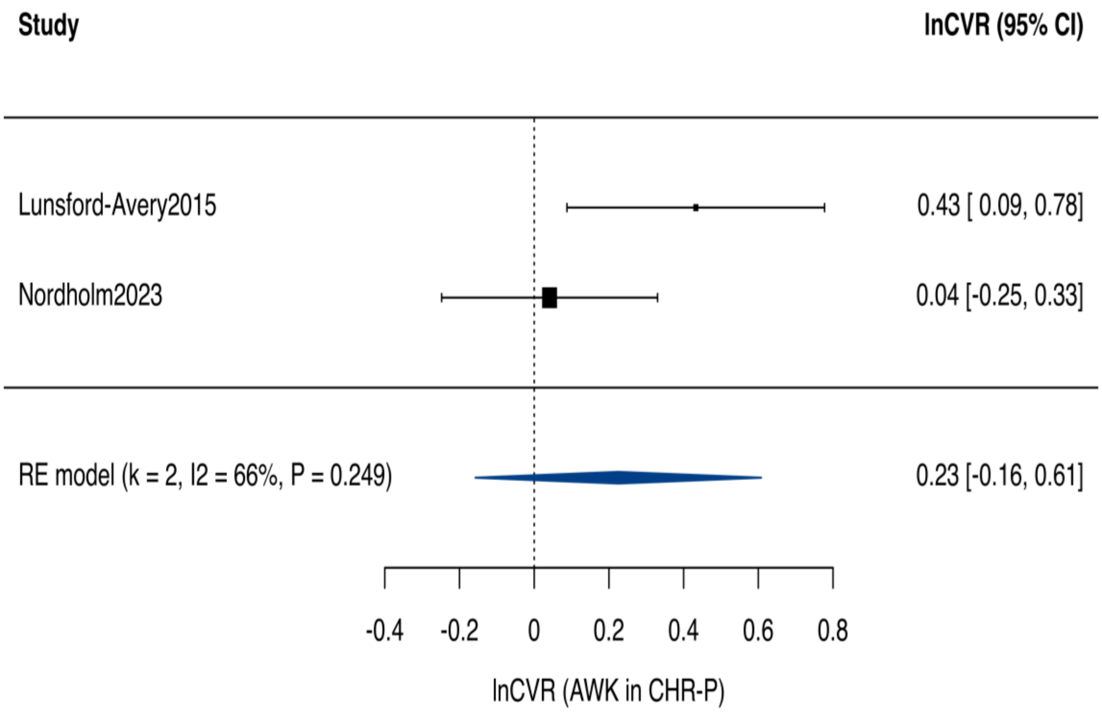

© 2026 Aronica R et al. *JAMA Network Open*.

eFigure 21. Number of Awakenings—SSD: lnVR (*k* = 3; REML)

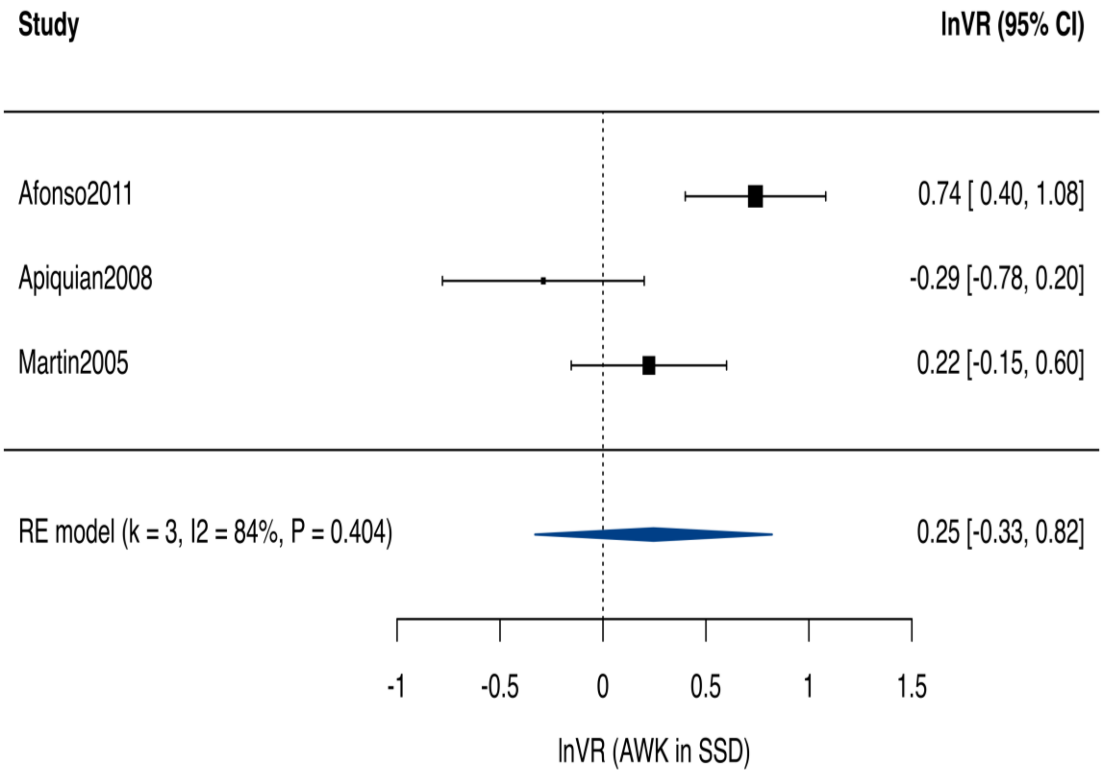

© 2026 Aronica R et al. *JAMA Network Open*.

eFigure 22. Number of Awakenings—SSD: lnCVR (*k* = 3; REML)

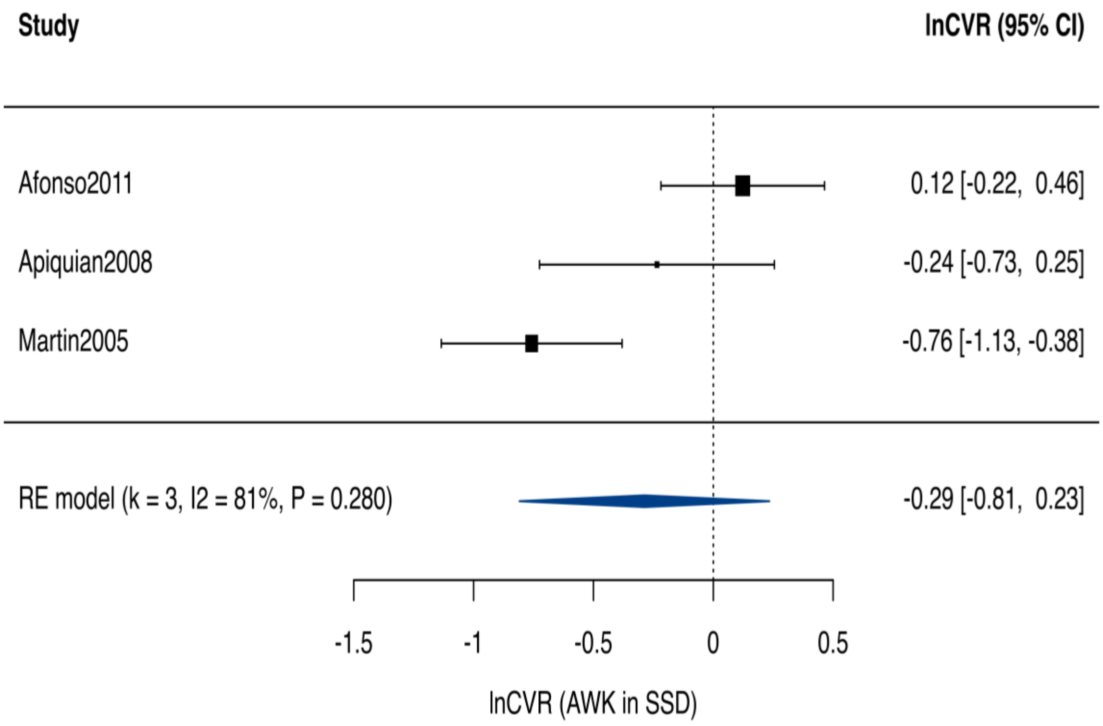

© 2026 Aronica R et al. *JAMA Network Open*.

eFigure 23. Wake After Sleep Onset (WASO)—CHR-P: lnVR (*k* = 4; REML)

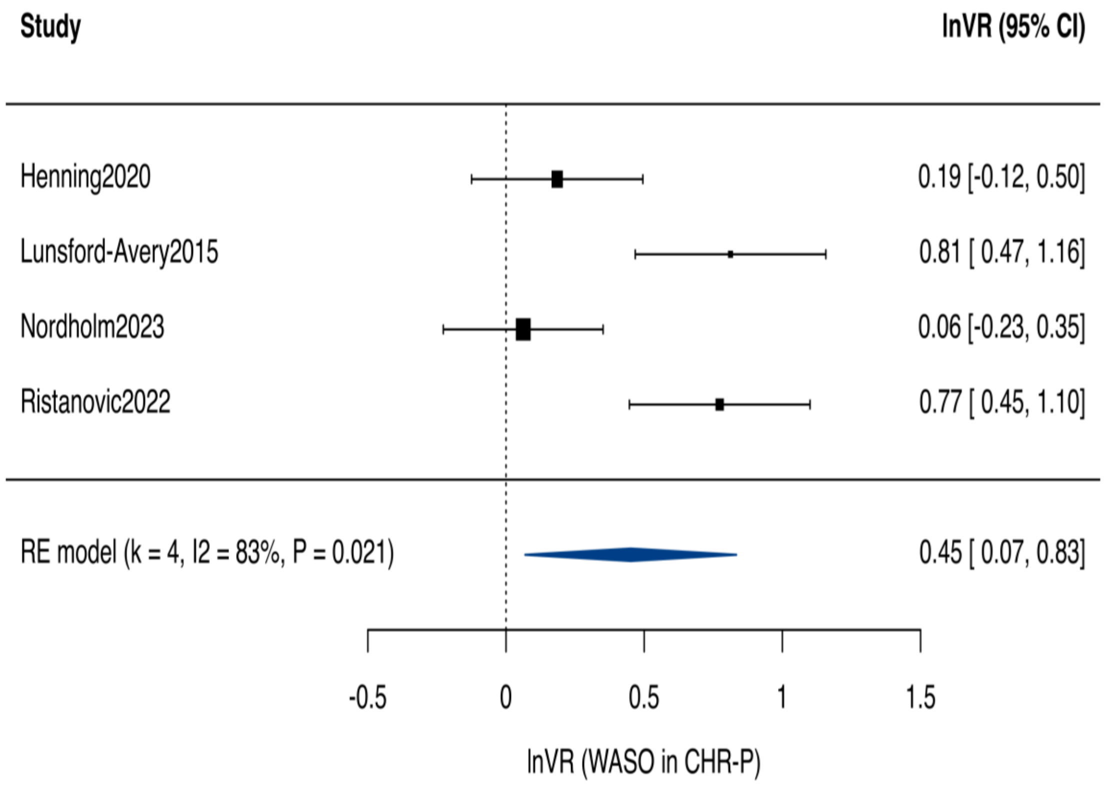

© 2026 Aronica R et al. *JAMA Network Open*.

eFigure 24. Wake After Sleep Onset—SSD: lnVR (*k* = 5; REML)

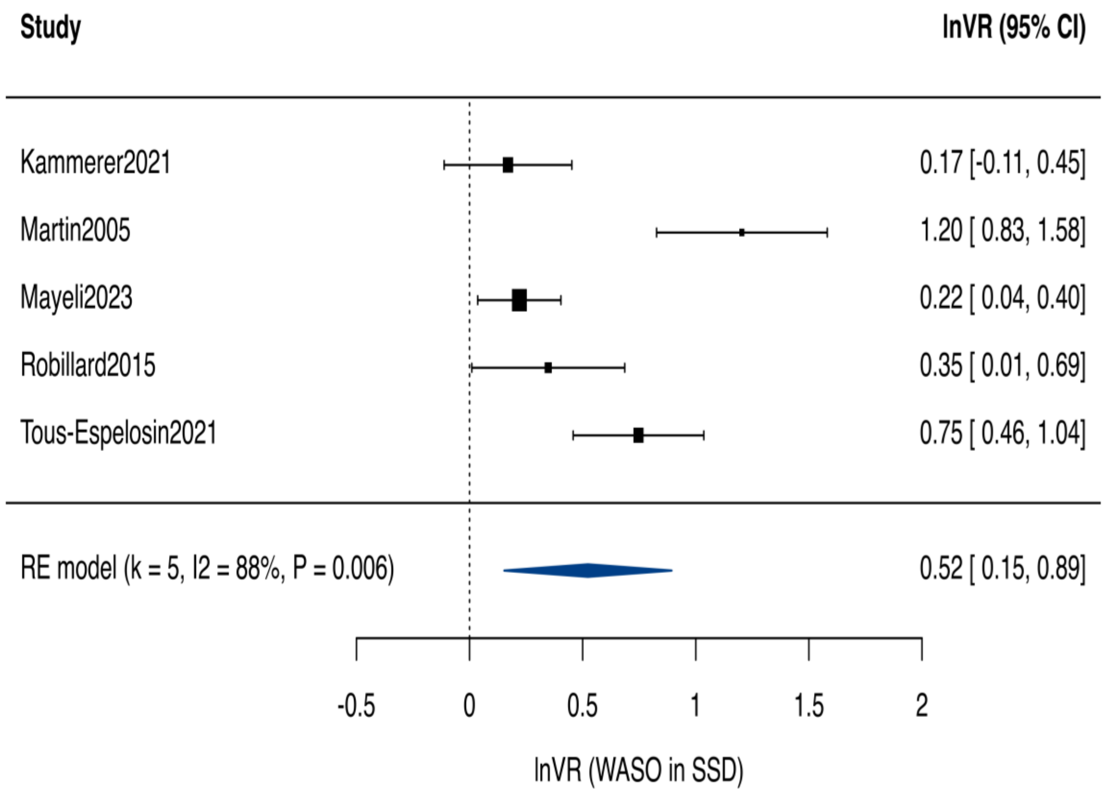

© 2026 Aronica R et al. *JAMA Network Open*.

eFigure 25. Wake After Sleep Onset—SSD: lnCVR (*k* = 5; REML)

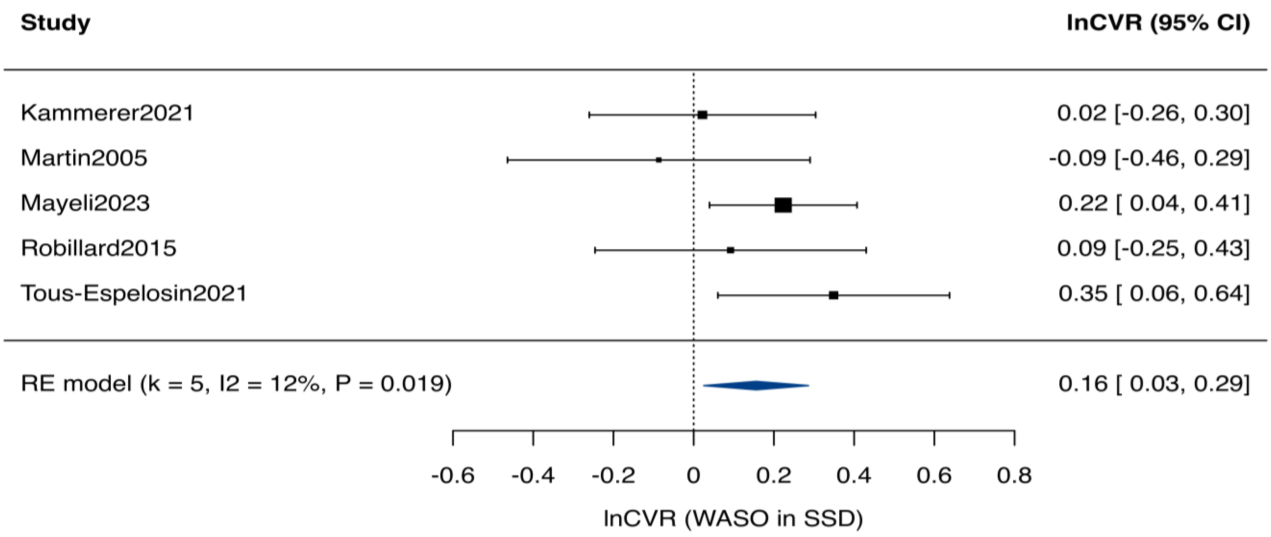

© 2026 Aronica R et al. *JAMA Network Open*.

eFigure 26. Sleep Efficiency (SE)—CHR-P: lnVR (*k* = 5; REML)

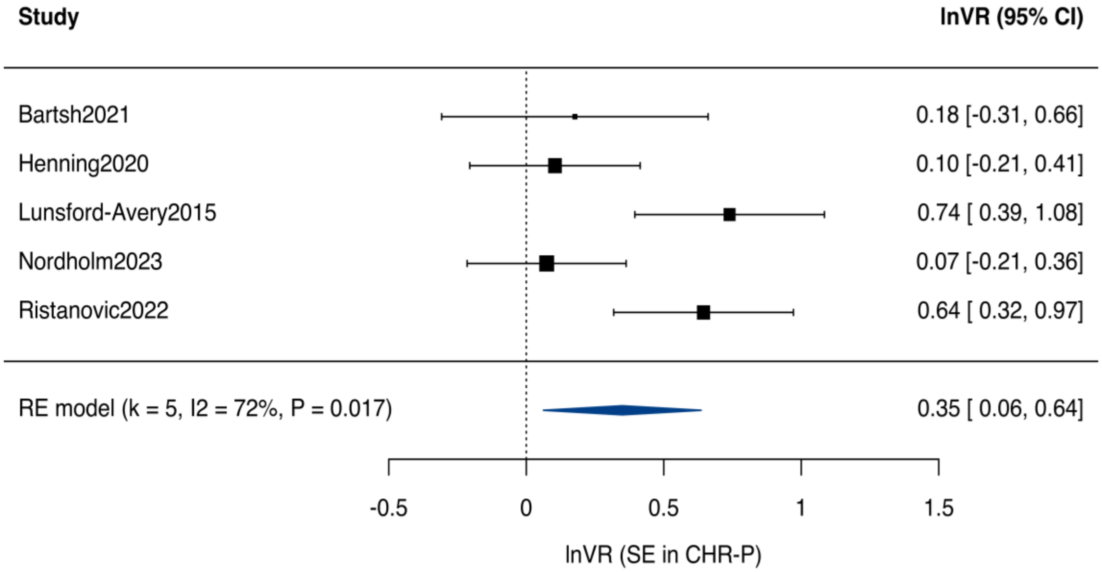

© 2026 Aronica R et al. *JAMA Network Open*.

eFigure 27. Sleep Efficiency—CHR-P: lnCVR (*k* = 5; REML)

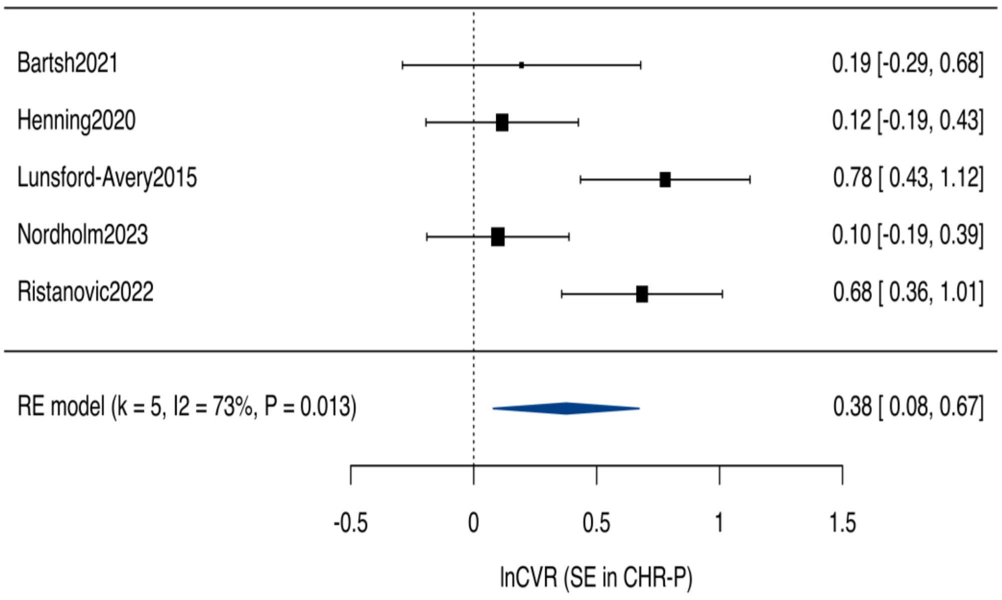

© 2026 Aronica R et al. *JAMA Network Open*.

eFigure 28. Sleep Efficiency—SSD: lnVR (*k* = 9; REML)

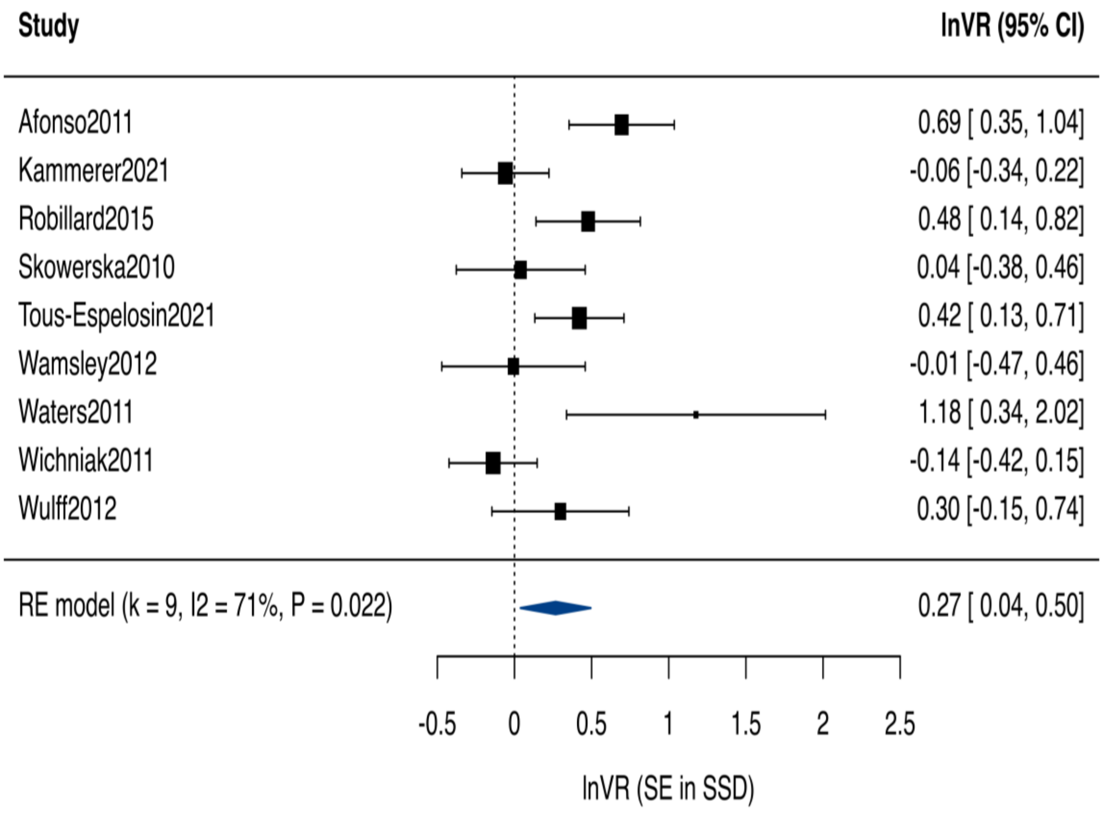

© 2026 Aronica R et al. *JAMA Network Open*.

eFigure 29. Sleep Efficiency—SSD: lnCVR (*k* = 9; REML)

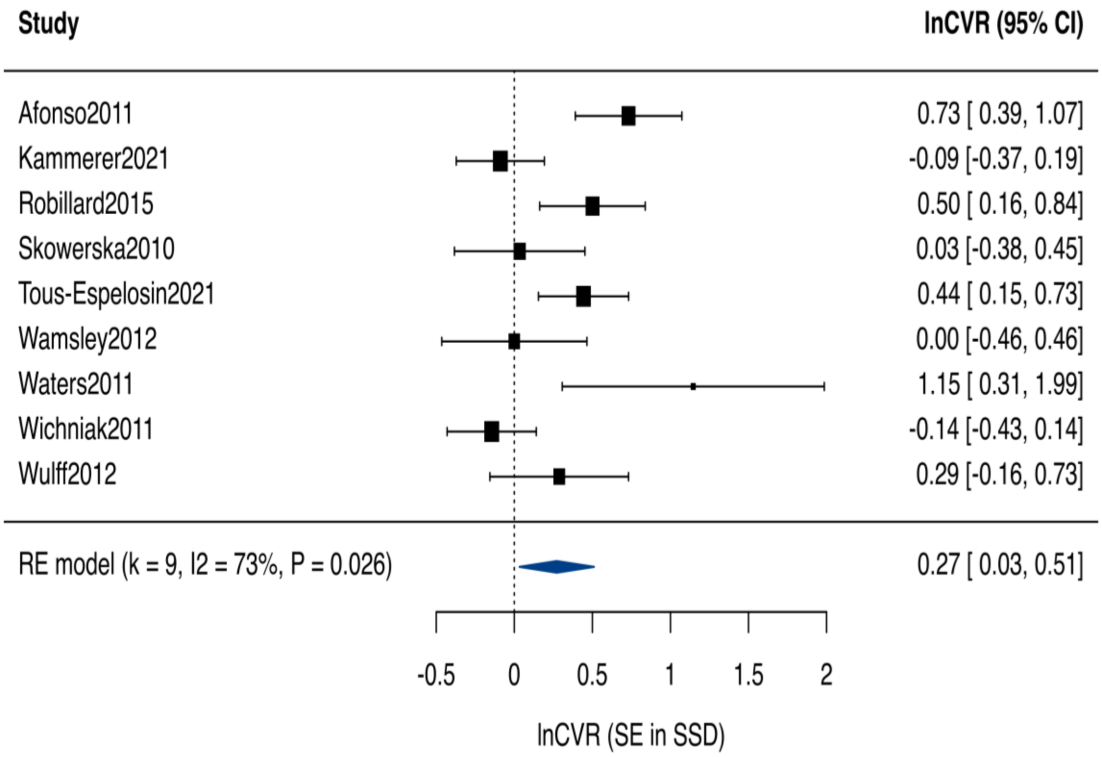

© 2026 Aronica R et al. *JAMA Network Open*.

eFigure 30. Leave-One-Out Sensitivity Analyses for Total Sleep Time (TST), Time in Bed (TIB), Wake After Sleep Onset (WASO), and Sleep Efficiency (SE) in Patients With Clinical High Risk for Psychosis (CHR-P) and Schizophrenia Spectrum Disorders (SSD)

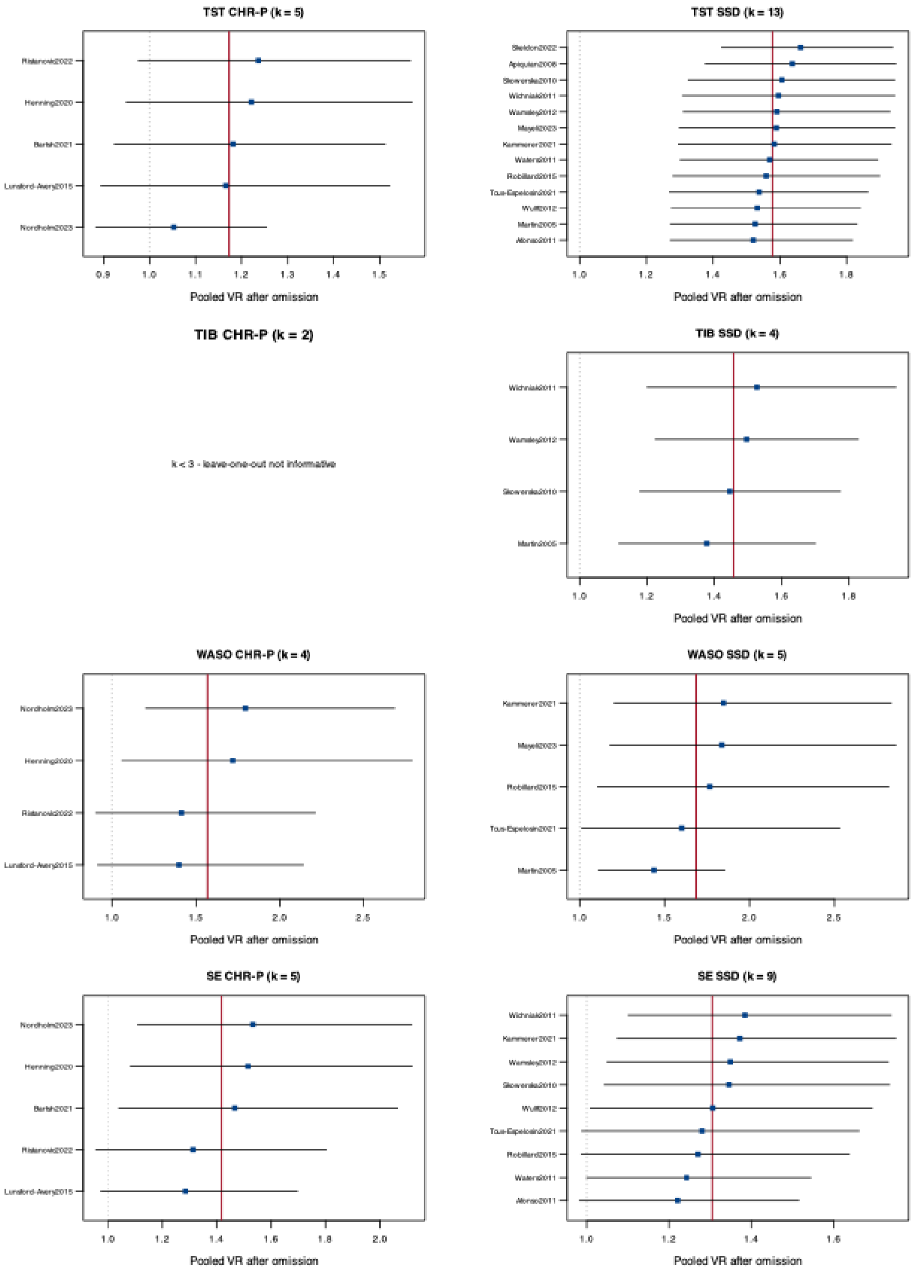

© 2026 Aronica R et al. *JAMA Network Open*.

eFigure 31. Baujat Plots for Total Sleep Time (TST), Time in Bed (TIB), Wake After Sleep Onset (WASO), and Sleep Efficiency (SE) in Patients With Clinical High Risk for Psychosis (CHR-P) and Schizophrenia Spectrum Disorders (SSD)

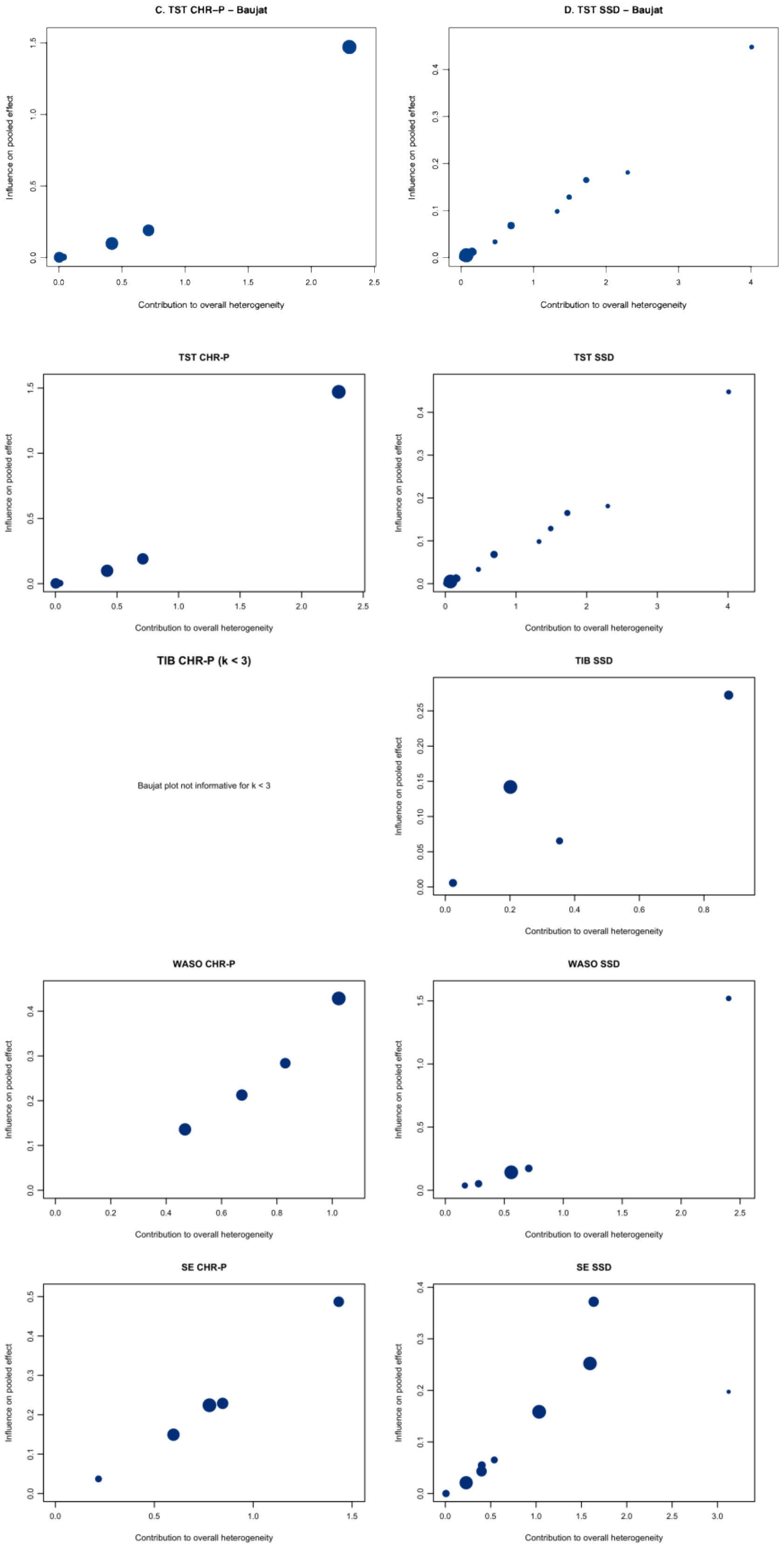

© 2026 Aronica R et al. *JAMA Network Open*.

eFigure 32. Funnel Plot and Egger Regression for the Primary Outcome (Total Sleep Time [TST] lnVR) in Patients With Schizophrenia Spectrum Disorders (SSD)

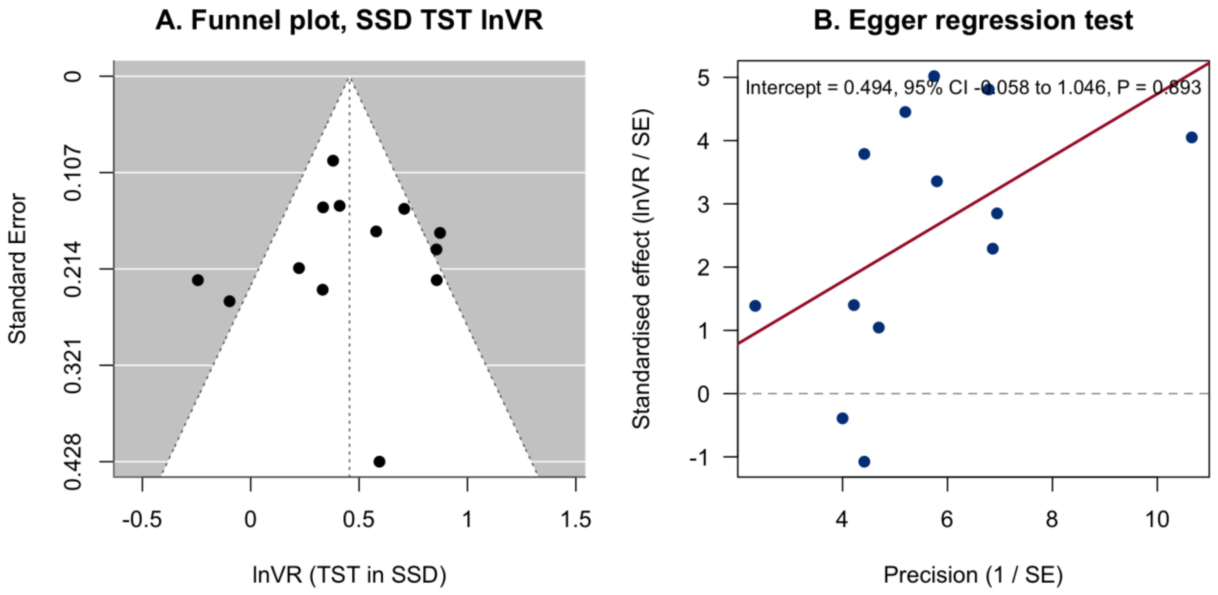

© 2026 Aronica R et al. *JAMA Network Open*.

eFigure 33. Funnel Plots for Key Secondary Outcomes (Time in Bed [TIB], Wake After Sleep Onset [WASO], and Sleep Efficiency [SE]) in Patients With Clinical High Risk for Psychosis (CHR-P) and Schizophrenia Spectrum Disorders (SSD)

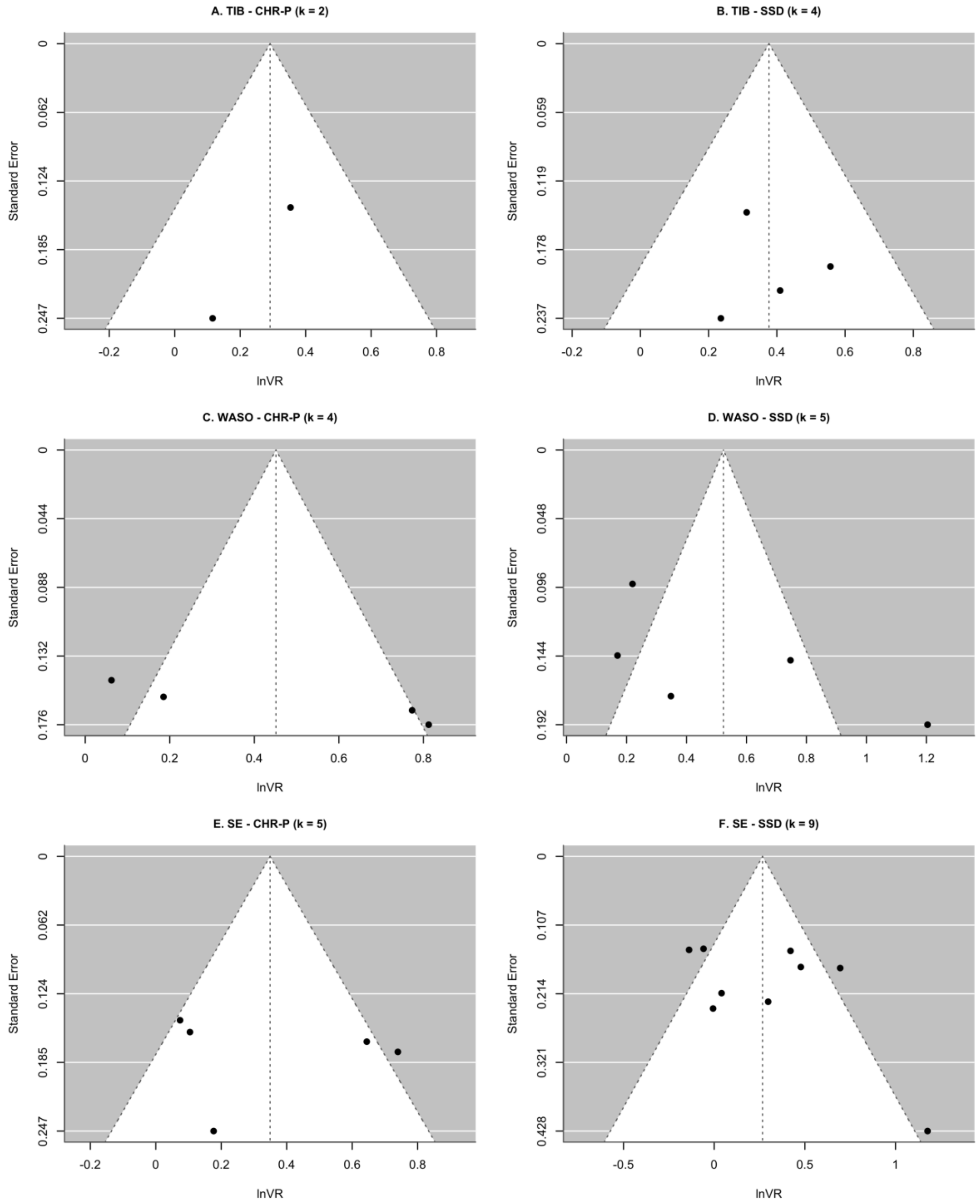

Caption. Funnel plots of lnVR against standard error for time in bed (panels A, B), wake after sleep onset (panels C, D) and sleep efficiency (panels E, F), separately for CHR-P (left column) and SSD (right column). Each panel's dashed vertical line indicates the random-effects pooled estimate. Egger regression was not performed for these comparisons because each had fewer than 10 contributing studies.

© 2026 Aronica R et al. *JAMA Network Open*.

eReferences

1. Afonso P, Figueira ML, Paiva T. Sleep-promoting action of the endogenous melatonin in schizophrenia compared to healthy controls. *International Journal of Psychiatry in Clinical Practice*. 2011;15(4):311-315. doi:10.3109/13651501.2011.605954
2. Apiquian R, Fresán A, Muñoz-Delgado J, Kiang M, Ulloa RE, Kapur S. Variations of rest – activity rhythm and sleep – wake in schizophrenic patients versus healthy subjects: An actigraphic comparative study. *Biological Rhythm Research*. 2008;39(1):69-78. doi:10.1080/09291010701318253
3. Bartsch U, Corbin LJ, Hellmich C, et al. Schizophrenia-associated variation at *ZNF804A* correlates with altered experience-dependent dynamics of sleep slow waves and spindles in healthy young adults. *SLEEP*. 2021;44(12):zsab191. doi:10.1093/sleep/zsab191
4. Hennig T, Schlier B, Lincoln TM. Sleep and psychotic symptoms: An actigraphy and diary study with young adults with low and elevated psychosis proneness. *Schizophrenia Research*. 2020;221:12-19. doi:10.1016/j.schres.2019.09.012
5. Kammerer MK, Mehl S, Ludwig L, Lincoln TM. Sleep and circadian rhythm disruption predict persecutory symptom severity in day-to-day life: A combined actigraphy and experience sampling study. *Journal of Abnormal Psychology*. 2021;130(1):78-88. doi:10.1037/abn0000645
6. Lunsford-Avery JR, LeBourgeois MK, Gupta T, Mittal VA. Actigraphic-measured sleep disturbance predicts increased positive symptoms in adolescents at ultra high-risk for psychosis: A longitudinal study. *Schizophrenia Research*. 2015;164(1-3):15-20. doi:10.1016/j.schres.2015.03.013
7. Martin JL, Jeste DV, Ancoli-Israel S. Older schizophrenia patients have more disrupted sleep and circadian rhythms than age-matched comparison subjects. *Journal of Psychiatric Research*. 2005;39(3):251-259. doi:10.1016/j.jpsychires.2004.08.011
8. Mayeli A, LaGoy AD, Smagula SF, et al. Shared and distinct abnormalities in sleep-wake patterns and their relationship with the negative symptoms of Schizophrenia Spectrum Disorder patients. *Mol Psychiatry*. 2023;28(5):2049-2057. doi:10.1038/s41380-023-02050-x
9. Nordholm D, Jensen MA, Glenthøj LB, et al. Sleep disturbances and the association with attenuated psychotic symptoms in individuals at ultra high-risk of psychosis. *Journal of Psychiatric Research*. 2023;158:143-149. doi:10.1016/j.jpsychires.2022.12.041
10. Ristanovic I, Haase CM, Lunsford-Avery JR, Mittal VA. The relationship between stress responding in family context and stress sensitivity with sleep dysfunction in individuals at clinical high-risk for psychosis. *Journal of Psychiatric Research*. 2022;149:194-200. doi:10.1016/j.jpsychires.2022.02.038

© 2026 Aronica R et al. *JAMA Network Open*.

11. Robillard R, Hermens DF, Naismith SL, et al. Ambulatory sleep-wake patterns and variability in young people with emerging mental disorders. *jpn*. 2015;40(1):28-37. doi:10.1503/jpn.130247

12. Skeldon AC, Dijk DJ, Meyer N, Wulff K. Extracting Circadian and Sleep Parameters from Longitudinal Data in Schizophrenia for the Design of Pragmatic Light Interventions. *Schizophrenia Bulletin*. 2022;48(2):447-456. doi:10.1093/schbul/sbab124

13. Skowerska A, Wichniak A, Skalski M. [Sleep and circadian rhythm disturbances in schizophrenia]. *Psychiatr Pol*. 2010;44(5):621-631.

14. Tous-Espelosin M, De Azua SR, Iriarte-Yoller N, et al. Clinical, physical, physiological, and cardiovascular risk patterns of adults with schizophrenia: CORTEX-SP study. *Psychiatry Research*. 2021;295:113580. doi:10.1016/j.psychres.2020.113580

15. Wamsley EJ, Tucker MA, Shinn AK, et al. Reduced Sleep Spindles and Spindle Coherence in Schizophrenia: Mechanisms of Impaired Memory Consolidation? *Biological Psychiatry*. 2012;71(2):154-161. doi:10.1016/j.biopsych.2011.08.008

16. Waters F, Sinclair C, Rock D, Jablensky A, Foster RG, Wulff K. Daily variations in sleep–wake patterns and severity of psychopathology: A pilot study in community-dwelling individuals with chronic schizophrenia. *Psychiatry Research*. 2011;187(1-2):304-306. doi:10.1016/j.psychres.2011.01.006

17. Wichniak A, Skowerska A, Chojnacka-Wójtowicz J, et al. Actigraphic monitoring of activity and rest in schizophrenic patients treated with olanzapine or risperidone. *Journal of Psychiatric Research*. 2011;45(10):1381-1386. doi:10.1016/j.jpsychires.2011.05.009

18. Wulff K, Dijk DJ, Middleton B, Foster RG, Joyce EM. Sleep and circadian rhythm disruption in schizophrenia. *Br J Psychiatry*. 2012;200(4):308-316. doi:10.1192/bjp.bp.111.096321
