# Supplementary material for: Interindividual Sleep Variability Across the Psychosis Spectrum: A Systematic Review and Meta-Analysis
Source: JAMA Netw Open. 2026 Jul 22;9(7):e2624358. doi: 10.1001/jamanetworkopen.2026.24358 (PMC13392811; doi:10.1001/jamanetworkopen.2026.24358)
Supplement: Supplement 2. — Data Sharing Statement [file jamanetwopen-e2624358-s002.pdf]

Data Sharing Statement

Aronica. Interindividual Sleep Variability Across the Psychosis Spectrum. *JAMA Netw Open*. Published July 22, 2026. doi:10.1001/jamanetworkopen.2026.24358

Data
Data available: No

Additional Information
Explanation for why data not available: All relevant data have been included in the original manuscript and online supplement. The analytic R script and the preregistered protocol are openly available on the Open Science Framework (https://osf.io/5n8bc/) under a CC-BY 4.0 license.
